# Biomimetic Grapefruit-Derived Extracellular Vesicles for Safe and Targeted Delivery of Sodium Thiosulfate against Vascular Calcification

**DOI:** 10.1021/acsnano.3c05261

**Published:** 2023-12-06

**Authors:** Weijing Feng, Yintong Teng, Qingping Zhong, Yangmei Zhang, Jianwu Zhang, Peng Zhao, Guoqing Chen, Chunming Wang, Xing-Jie Liang, Caiwen Ou

**Affiliations:** † The Tenth Affiliated Hospital of Southern Medical University (Dongguan People’s Hospital), Southern Medical University or The First School of Clinical Medicine, Southern Medical University, Dongguan 523018, China; ‡ Department of Cardiology, State Key Laboratory of Organ Failure Research, Guangdong Provincial Key Laboratory of Cardiac Function and Microcirculation, Nanfang Hospital, Southern Medical University, Guangzhou 510515, China; § NMPA Key Laboratory for Research and Evaluation of Drug Metabolism, Guangdong Provincial Key Laboratory of New Drug Screening, Guangdong Provincial Key Laboratory of Cardiac Function and Microcirculation, School of Pharmaceutical Sciences, Southern Medical University, Guangzhou 510515, China; ∥ Cardiology Department of Panyu Central Hospital and Cardiovascular Disease Institute of Panyu District, Guangzhou 511400, China; ⊥ Institute of Chinese Medical Sciences & State Key Laboratory of Quality Research in Chinese Medicine, University of Macau, Macau 00000, SAR, China; # Chinese Academy of Sciences (CAS) Center for Excellence in Nanoscience and CAS Key Laboratory for Biomedical Effects of Nanomaterials and Nanosafety, National Center for Nanoscience and Technology, Beijing 100190, China

**Keywords:** plant derived-extracellular vesicles, biomimetic delivery system, biomimetic nanodrugs, vascular calcification treatment, sodium thiosulfate, bone-vascular axis

## Abstract

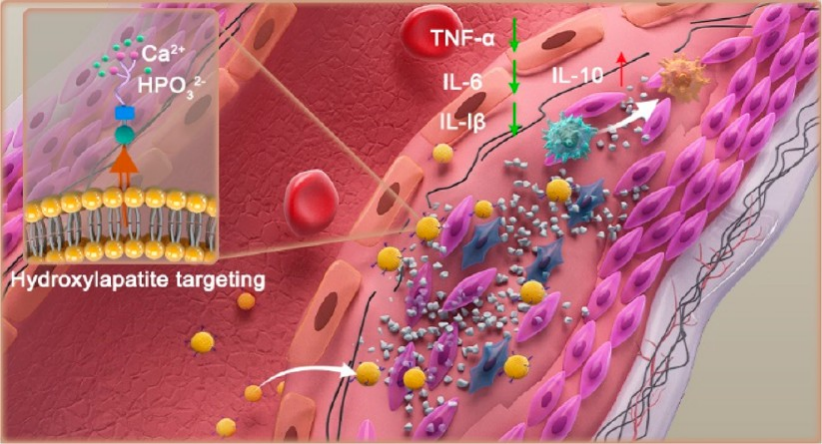

As the prevalence of vascular calcification (VC), a strong contributor to cardiovascular morbidity and mortality, continues to increase, the need for pharmacologic therapies becomes urgent. Sodium thiosulfate (STS) is a clinically approved drug for therapy against VC; however, its efficacy is hampered by poor bioavailability and severe adverse effects. Plant-derived extracellular vesicles have provided options for VC treatment since they can be used as biomimetic drug carriers with higher biosafety and targeting abilities than artificial carriers. Inspired by natural grapefruit-derived extracellular vesicles (EVs), we fabricated a biomimetic nanocarrier comprising EVs loaded with STS and further modified with hydroxyapatite crystal binding peptide (ESTP) for VC-targeted delivery of STS. *In vitro*, the ESTP nanodrug exhibited excellent cellular uptake capacity by calcified vascular smooth muscle cells (VSMCs) and subsequently inhibited VSMCs calcification. In the VC mice model, the ESTP nanodrug showed preferentially the highest accumulation in the calcified arteries compared to other treatment groups. Mechanistically, the ESTP nanodrug significantly prevented VC via driving M2 macrophage polarization, reducing inflammation, and suppressing bone-vascular axis as demonstrated by inhibiting osteogenic phenotype trans-differentiation of VSMCs while enhancing bone quality. In addition, the ESTP nanodrug did not induce hemolysis or cause any damage to other organs. These results suggest that the ESTP nanodrug can prove to be a promising agent against VC without the concern of systemic toxicity.

## Introduction

Vascular calcification (VC) is prevalent in patients with hypertension,^1^ diabetes mellitus,^2,3^ and chronic kidney disease,^4^ which remains an important public health issue as a leading contributor to adverse cardiovascular events worldwide.^3,5^ Up to now, surgical and endovascular strategies are still the mainstays in the treatment of VC; however, their effects on the cardiovascular outcomes of patients are still far from satisfactory.^6,7^ Although agents, such as phosphate binders and calcimimetic agents, have been reported to attenuate the progression of VC at the early stage,^7^ most patients miss the best time for treatment because VC identified by a computed tomography (CT) scan is usually too late to intervene and even can not tolerate their side effects.^8,9^ Considering the limited efficacy of these treatments for VC and their considerable adverse effects, it is urgent to explore strategies for VC treatment, with improved overall efficacy and less toxicity.^7^

Sodium thiosulfate (STS) has been used to deal with calciphylaxis in clinic and provided potential opportunity against various ectopic calcification including VC.^10−12^ Findings from experimental studies suggested that STS attenuated VC possibly due to its potent chelating and antioxidant properties.^13,14^ However, recent clinical evidence from GIPS-IV study reported that STS did not improve cardiovascular outcomes in patients with myocardial infarction^15^ because most patients can not tolerate high doses of STS due to offensively strong odor and adverse reactions such as nausea and vomiting.^15^ In addition, traditional systemic administration of STS has been associated with severe adverse effects in normal organs due to undesirable accumulation in normal tissues such as the reduction of bone mineral density.^13,16^ Moreover, only a small fraction of STS could actually reach the site of VC.^16^ Consequently, an ideal therapeutic strategy should involve efficient delivery of STS and durable and specific anticalcification treatment together with minimized adverse events and less toxicity with essential dose.

Extracellular vesicles (EVs), including microvesicles, apoptotic bodies, and exosomes, are lipid bilayer particles secreted by cells. Although mammalian cell-derived EVs have been regarded as a promising platform for drug delivery, there are still many obstacles to their clinical translations, including cancer-stimulating risk,^17^ rapid blood clearance, and limited yields.^18−20^ Recently, more attention has been moved to plant-derived EVs, which were found in various plants and have similar physical and chemical properties to mammalian-derived EVs.^21^ In contrast to mammalian-derived EVs (such as milk EVs, *etc*.) and other synthetic drug carriers, plant-derived EVs have excellent properties, such as but not limited to sufficient plant resources, higher yield,^22^ lower immunological risk,^23^ and without any zoonotic or human pathogens.^24^ Compared to liposomes, plant-derived EVs are more suitable as intravascular drug carriers due to lower risk of thrombosis and avoidable toxicity from residual organic compounds.^23,25,26^ Additionally, compelling evidence reported that plant-derived EVs not only inherit the natural bioactive functions of source plants but also possess great potential in drug delivery attributable to their specific structure.^27^ The lipid bilayer structure of plant-derived EVs enables them to effectively encapsulate hydrophilic and hydrophobic drugs and improves their stability, solubility, and bioavailability.^28^ Of note, EVs derived from various plants, such as grapefruit,^29^ ginger,^30^ ginseng,^31^ lemon, and so on,^32^ have been demonstrated for their obvious effects in treating inflammatory bowel diseases and tumors. However, the influence of plant-derived EVs on VC and efficient strategies for carrying therapeutic drugs to prevent VC are still unknown.

**Figure 1:**
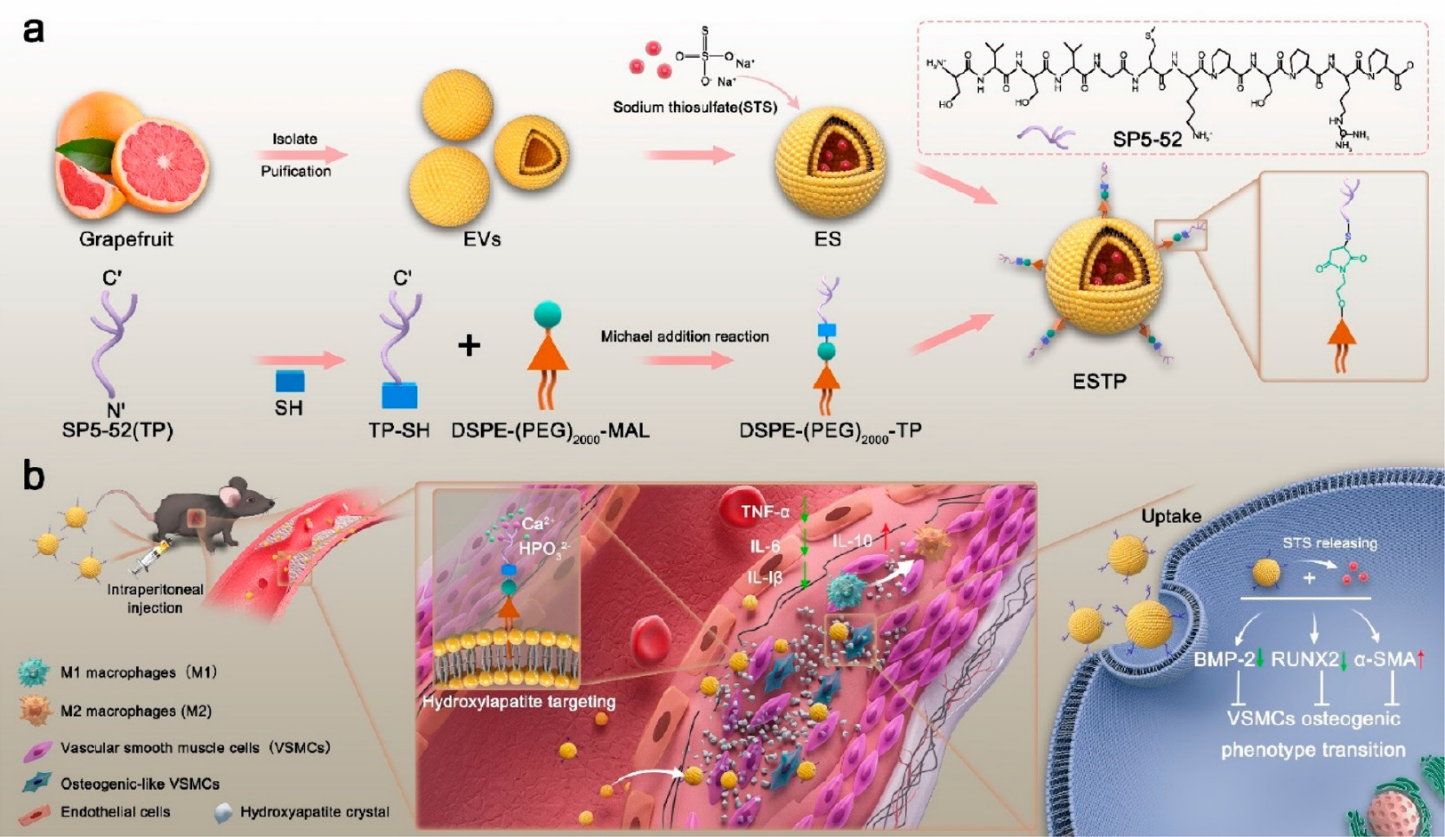
**Schematic illustration of ESTP for suppressing vascular calcification**. (a) Grapefruit-derived extracellular vesicle (EVs) nanodrugs (marked with ESTP) were fabricated by loading sodium thiosulfate (STS) in EVs and then modifying hydroxyapatite-binding peptide SP5–52-SH (TP) onto the surface of EVs using DSPE-(PEG)2000-maleimide as a linker. (b) After systemic administration, ESTP nanodrugs actively accumulate in the sites of vascular calcification (VC) with the help of TP, wherein not only the release of STS but also the natural bioactive compounds of EVs effectively reduce inflammation via driving M2 macrophage polarization and inhibiting osteogenic phenotype trans-differentiation of vascular smooth muscle cells (VSMCs). The synergism of natural grapefruit-derived EVs, clinically approved drug STS, and TP results in potent anti-VC efficacy with minor side effects.

Herein, we constructed a natural grapefruit-derived EVs-based drug delivery system that enabled the active to actively accumulate in the site of VC, achieving highly efficient attenuation of the development of VC. As is well-known, VC, especially at the early stage, is characterized by vascular smooth muscle cells (VSMCs) phenotypic transformation from contractile into osteogenic phenotype and subsequently leading to hydroxyapatite (HA) deposition in the blood vessels.^5,33^ Inspired by this, we engineered HA binding peptide SP5–52-SH (TP) onto the surface of grapefruit-derived EVs to deliver the water-soluble clinically approved drug STS for the fabrication of TP-EVs-STS (ESTP) (Figure 1a). The introduction of TP enabling ESTP has effective accumulation in VC and excellent cellular uptake capacities in calcified VSMCs. After systemic administration, the as-prepared ESTP nanodrugs actively accumulate in VC owing to the binding capacity of TP, wherein not only the release of STS but also the natural bioactive compounds of EVs effectively reduce inflammation via driving M2 macrophage polarization and suppressing bone-vascular axis as demonstrated by inhibiting osteogenic phenotype trans-differentiation of VSMCs while enhancing bone quality. The synergism of natural grapefruit-derived EVs, clinically approved drug STS, and TP results in potent anti-VC efficacy with minor side effects (Figure 1b). This natural grapefruit-derived EVs-based biomimetic drug delivery system is a promising candidate for VC treatment, providing potential strategies to use natural grapefruit-derived EVs as biocompatible drug delivery nanoplatforms for cardiovascular disease treatment.

## Results/Discussion

### Characterization of Grapefruit-Derived ESTP Nanodrugs

**Figure 2:**
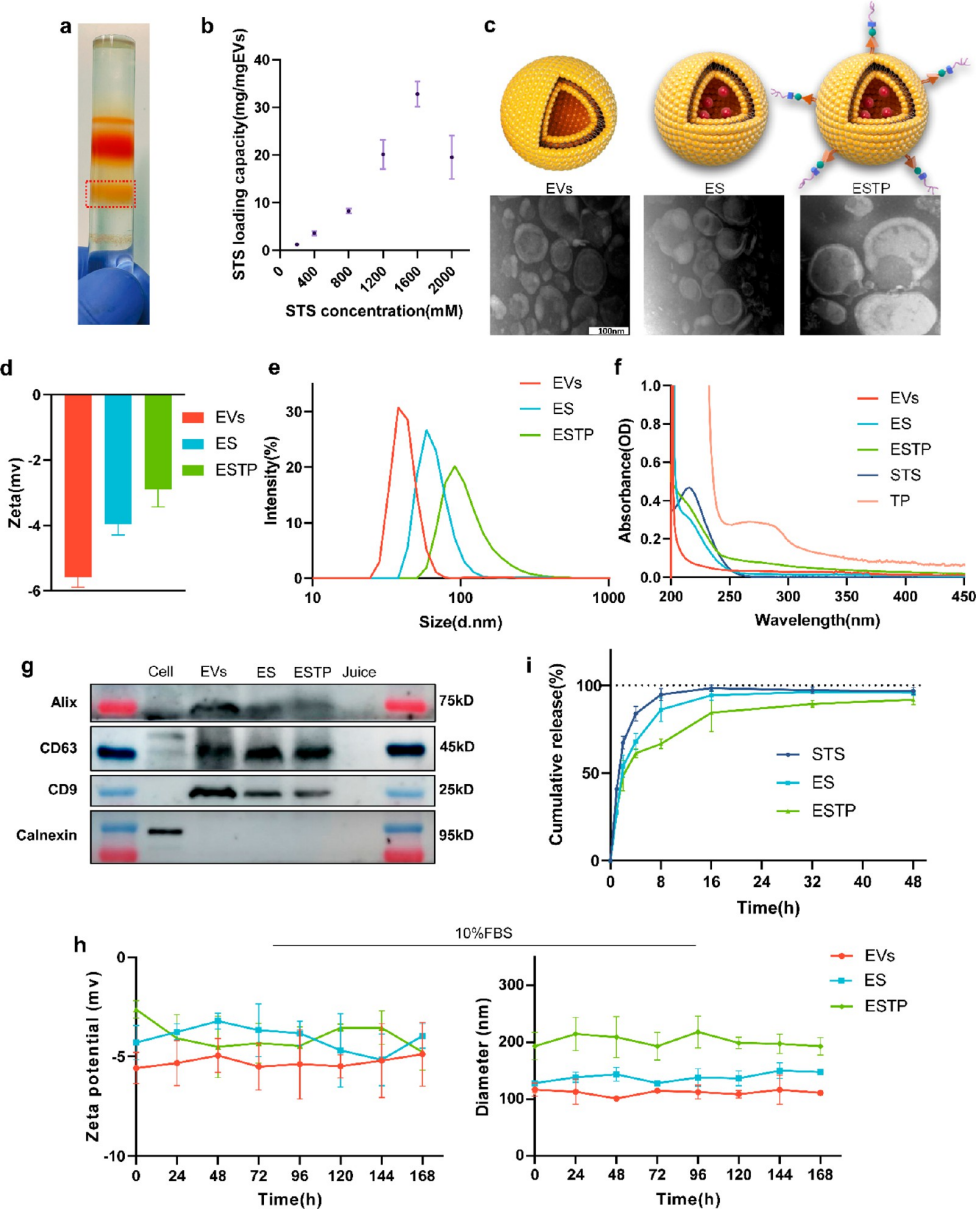
**Characterization of grapefruit-derived EVs, ES, and ESTP nanodrugs**. (a) Grapefruit-derived EVs were purified by sucrose density gradient (8%/30%/45%/60%) under ultracentrifugation. The interface of 30%/45% (as marked in red rectangle) was harvested and noted as EVs for further use. (b) Comparison of STS loading efficiency in EVs between different STS feed concentration. Loading efficiency of STS in ES was calculated by quantifying the concentration of STS in supernatant before and after ultracentrifugation using UV–vis at the wavelength of 215 nm. (c) Transmission electron microscopy (TEM) images of grapefruit-derived EVs, ES, and ESTP. Scale bar:100 nm. (d) Zeta potential and (e) size distribution of EVs, ES, and ESTP measured by dynamic light scattering (DLS). (f) UV–vis spectra of grapefruit-derived EVs, ES, ESTP, STS, and TP. (g) The expression of Alix, CD9, CD63, and Calnexin of EVs, ES, and ESTP as determined by Western blotting using vascular smooth muscle cells (VSMCs) lysates as control. (h) The stability of EVs, ES, and ESTP in 10% FBS at 37 °C at different time points. (i)The cumulative release profile of STS from free STS and ES in PBS (pH 7.4) at 37 °C condition. Data are mean ± SEM (*n* = 3).

Plant-derived EVs can be developed as outstanding therapeutic carriers due to their low immunogenicity, easy accessibility, and good surface modifiability. Especially, grapefruit has been reported to exhibit potential health benefits in cardiovascular disease. Thus, grapefruit-derived EVs were selected to fabricate a drug delivery system toward VC treatment. In brief, EVs were isolated and purified from fresh juice (Figure 2a), and STS was encapsulated into EVs to prepare EVs-STS (ES). Then, TP was modified onto the surface of EVs using 1,2-distearoyl-sn-glycero-3-phosphoethanolamine (DSPE)-(PEG)_2000_-maleimide (PEG = poly(ethylene glycol)) as a linker to fabricate TP-EVs-STS (ESTP) nanodrugs which can selectively target HA for the identification of microcalcified plaque found in the vessel wall (Figure S1).^9,34^ To obtain the optimized ratio of STS and EVs, we measured the loading efficiency by varying different concentrations of STS using UV–vis analysis (Figure S2), and then we determined the STS concentrations of 1600 mM·L^–1^ as an optimized formulation to synthesize ES for the following experiments (Figure 2b). Similarly, we employed the TP/ES (m/m) of 4/1 as optimal feed ratio on the basis of the rate of combination and binding capacity of TP with ES (Figure S3a,b), characterization (Figure S3c,d) of ESTPs, and the effect of different products on mouse VSMCs calcification models (Figure S4). In addition, free TP with different contents had no obvious influence on the mouse VSMCs calcification models (Figure S5). The STS loading weight and TP binding capacity in 1 mg of ESTPs were 31.6 ± 2.3 and1.9 ± 0.8 mg. Transmission electron microscopy (TEM) images showed typical vesicle morphology of EVs, ES, and ESTP with lipid bilayers (Figure 2c). The zeta potentials of STS, EVs, ES, and ESTP are shown in Figure 2d. The size distributions of EVs, ES, and ESTP were 113.4 ± 8.9, 137.1 ± 10.8, and 195.5 ± 18.2 nm, respectively (Figure 2e). UV–vis showed that ESTP has similar characteristic absorption peaks at 215 and 250 nm, which were highly consistent with the absorption peaks of STS and TP, respectively, suggesting that ESTP was fabricated as expected (Figure 2f). The results from Western blotting showed ESTP was enriched in protein markers of EVs (Alix, CD9 and CD63) and the lack of endoplasmic reticulum marker (Calnexin) was similar to the purified grapefruit-derived EVs, indicating that the introduction of STS and TP did not change the biological properties of ESTP (Figure 2g). In addition, to evaluate the stability of obtained ESTP under storage conditions in phosphate-buffered saline (PBS) (pH 7.4) at 4 °C, the size distribution and Zeta potential were measured, and no significant changes were observed within 30 days (Figure S6). Furthermore, we selected 10% fetal bovine serum (FBS) solution to mimic blood conditions, and the results presented that the size distribution and Zeta potential of EVs, ES, and ESTP had no significant changes within 168 h (Figure 2h). These data suggested that ESTP has excellent stability *in vitro* and *in vivo*. We then explored the release behavior of STS and ES in PBS (pH = 7.4) under 37 °C conditions (Figure 2i). It shown that in the presence of EVs shell, the drug release rate was lower than free STS in the first 8 h under physiological condition.

### *In Vitro* Biological Activity of ESTP

**Figure 3:**
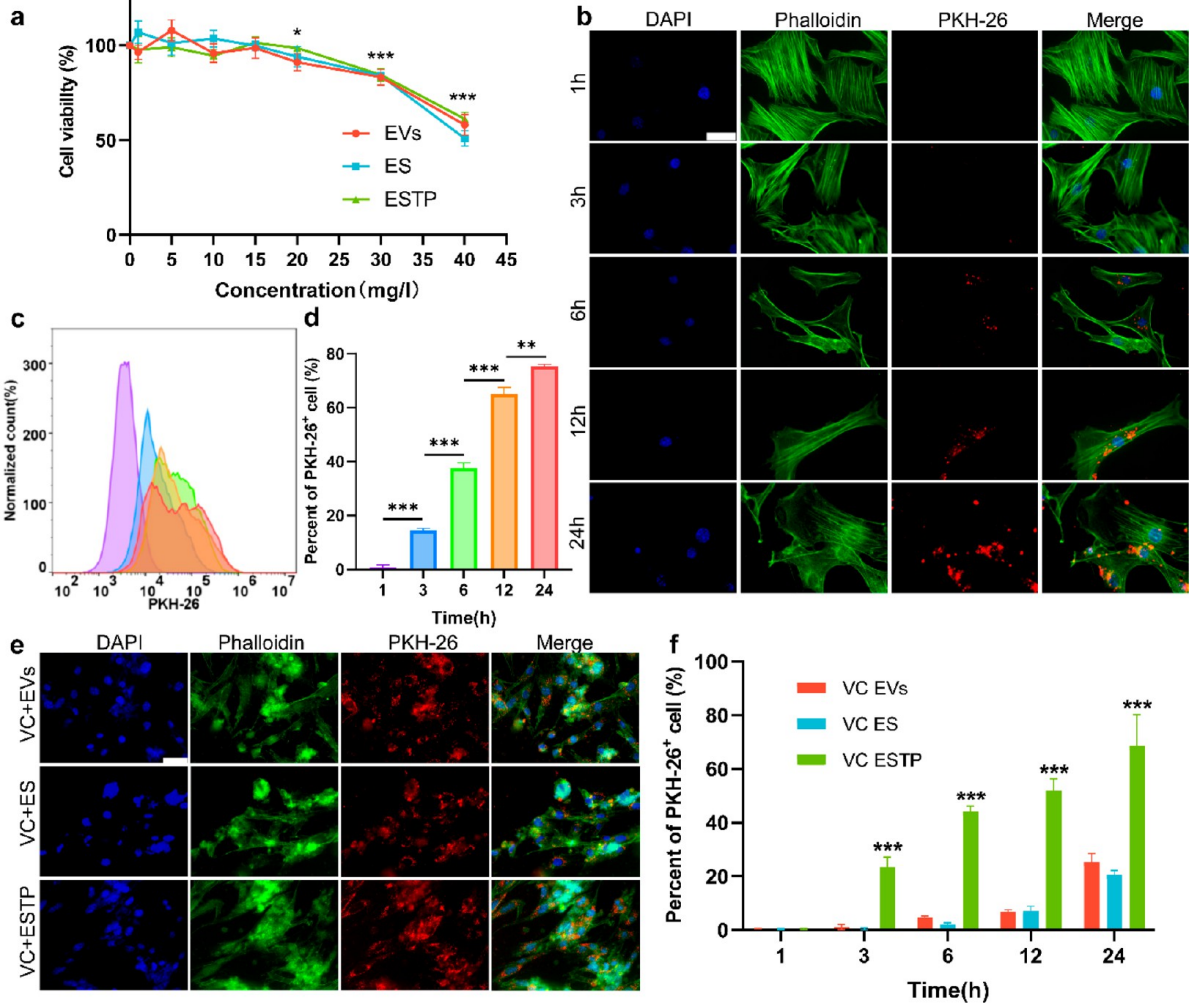
***In vitro*****biological activity of ESTP**. (a) Cell viability of primary mouse VSMCs treated with EVs, ES, and ESTP for 48 h (*n* = 5). (b) Representative immunofluorescence imaging of subcellular localization of PKH26-labeled EVs (red channel) incubated with primary mouse VSMCs for 1, 3, 6, 12, and 24 h. Phalloidin-FITC (green channel) and DAPI (blue channel) indicated the distribution of F-actin and the nucleus of VSMCs, respectively (*n* = 5). Scale bar: 50 μm. (c) Cellular uptake of PKH26-labeled EVs in primary mouse VSMCs for 24 h, as detected by (d) flow cytometry. (e) Representative immunofluorescence imaging of PKH26-labeled EVs, ES, and ESTP taken up by calcified VSMCs at 24 h (*n* = 3). Scale bar: 50 μm. (f) Flow cytometry analysis of cellular uptake of PKH26-labeled EVs, ES, and ESTP in calcified VSMCs at different time points (*n* = 5). Data are presented as mean ± SD *P < 0.05, **P < 0.01, ***P < 0.001.

To investigate the effect of nanodrugs on the viability of primary mouse vascular smooth muscle cells (VSMCs), VSMCs were exposed by different concentrations of STS, EVs, ES, and ESTP for 48 h. Cell counting kit-8 assays showed no obvious reduction in cell viability after adding STS at the concentration of <4 mM·L^–1^ or nanodrugs (EVs, ES, and ESTP) at the concentration of <20 mg L^–1^ (Figure 3a, Figure S7). Previous studies have proved that plant-derived EVs were internalized via a phagocytosis pathway by various cells.^30,35,36^ To detect the cellular uptake of EVs, VSMCs were incubated with PKH26-labeled EVs (red channel) for 1, 3, 6, 12, and 24 h and then labeled with phalloidin-fluorescein isothiocyanate (FITC) (green channel) and 4,6-diamidino-2–2-phenylindole (DAPI) (blue channel) to display the distribution of F-actin and nucleus (Figure 3b). Representative images from fluorescence microscopy exhibited that the PKH26-EVs with red fluorescence were mainly localized in the cytoplasm, and quantitative analysis of flow cytometry showed that cellular uptake of EVs increased with incubation time and the peak at 24 h point (Figure 3c,d). We supposed that ESTP could exhibit better cellular uptake ability in the mouse VSMCs calcification model due to the strong interaction between positively charged *-NH*_3_^*+*^ of TP and negatively charged *-HPO*_3_^2–^ of HA. To test this hypothesis, mouse VSMCs were modeled and incubated with PKH26-labeled EVs, ES, and ESTP. The results showed that compared to EVs and ES, the ESTP presented the best intracellular uptake capacity (Figure 3e,f, Figure S8). It suggested that the introduction of TP could obviously increase intracellular uptake due to enhanced affinity to HA in the calcified deposition.

### ESTP Nanodrugs Effectively Inhibited VSMCs Calcification *In Vitro*

**Figure 4:**
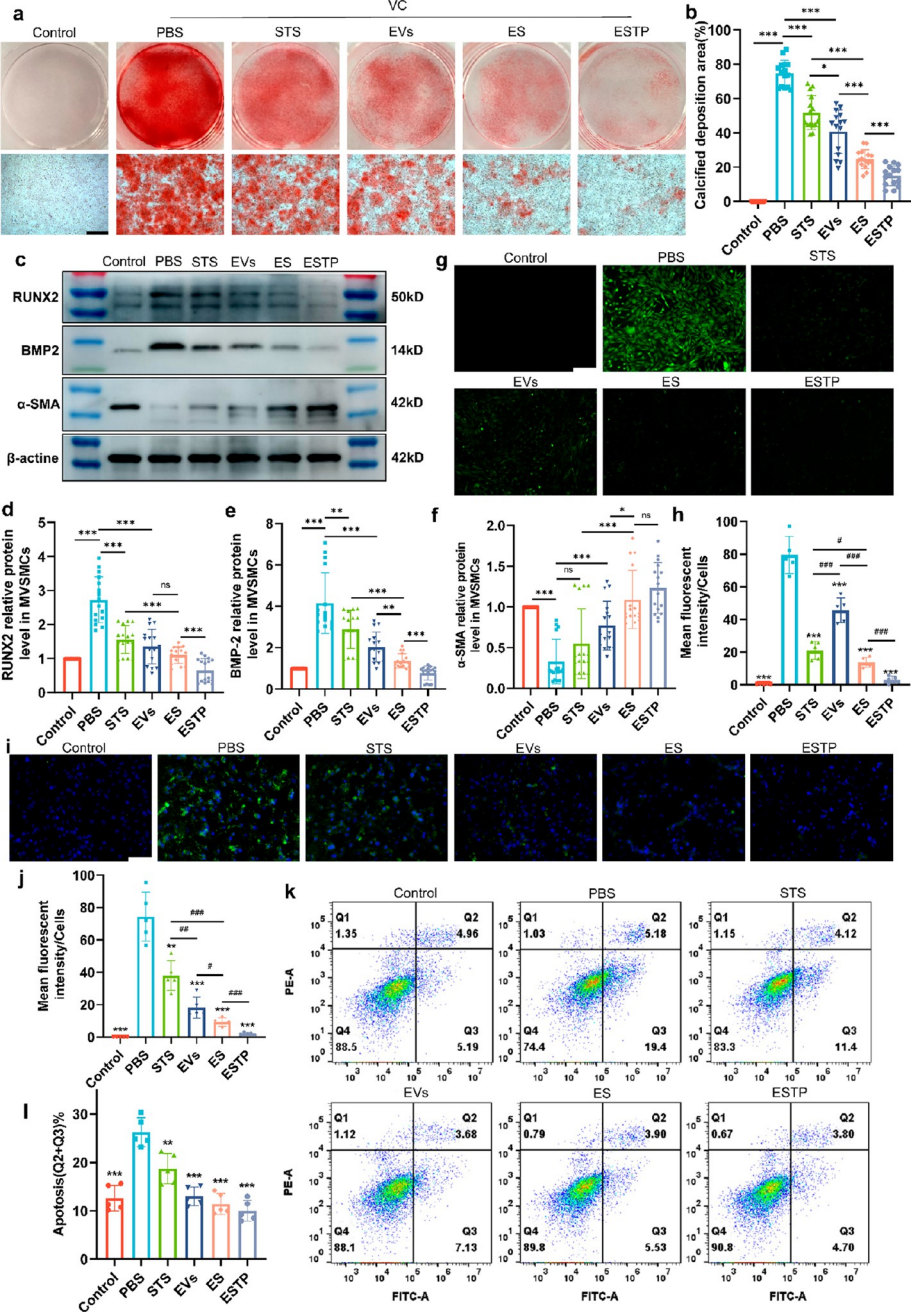
***In vitro*****effect of ESTP on mouse VSMCs calcification**. (a) Representative Alizarin red S staining of calcium nodule formation and the (b) quantification of calcium deposition in primary mouse VSMCs with PBS, STS, EVs, ES, or ESTP treatment under CM for 7 days. Scale bar: 500 μm. (c) Representative Western blot analysis and quantification of the (d, e) osteogenic marker (RUNX2 and BMP2) and (f) contractile marker (α-SMA) in mouse VSMCs after different treatments for 4 days. (g) ROS tracking and (h) quantification fluorescence analysis in CM-induced living cells after incubating with different treatments for 2 days. Green: DCFH-DA (with 488 nm laser). Scale bar: 200 μm. (i) Fluorescence images and (j) quantitative analysis of apoptosis assay in calcified VSMCs with different treatment for 2 days. Scale bar: 100 μm. Blue: DAPI-stained nucleus, green: TUNEL-labeled terminal deoxynucleotidyl transferase dUTP nick end (with 488 nm laser). (k) flow cytometry results and (l) quantification of cell apoptosis in calcified VSMCs from each group. *P* value style: *P < 0.05; **P < 0.01; or ***P < 0.001. Data are presented as mean ± standard deviation (SD) from five independent replicates.

To further evaluate the effects of STS or EVs on VC *in vitro*, primary mouse VSMCs were treated with different concentrations of STS (1, 2, or 4 mM·L^–1^) or EVs (5, 10, or 20 mg·L^–1^) in the presence of calcifying medium (CM) for 7 days. The results showed that the calcified deposition was reduced by STS or EVs in a dose-dependent manner (Figure S9). In order to exhibit better therapeutic effect of ESTP on VSMCs calcification models, 1 mM·L^–1^ of STS and 5 mg L^–1^ of EVs were chosen for subsequent cell experiments despite they cannot reach the optimal efficiency of inhibition of calcification when used alone.^37^ Alizarin Red S staining showed that ESTP has the best anticalcification effect on primary VSMCs compared to other treatment groups (PBS, STS, EVs, and ES) (Figure 4a,b). Of this, the anticalcification effect of ESTP was more marked than ES, which was possibly attributed to the better cellular uptake ability of ESTP for VSMCs calcification models. Osteogenic phenotype trans-differentiation of VSMCs is a critical process in the development of vascular calcification. Next, we detected the expression of contractile and osteogenic phenotypes in VSMCs with different treatments. The results from Western blot analysis showed that the expression of contractile phenotype marker (α-SMA) significantly increased, whereas the osteogenic phenotype markers (RUNX2 and BMP2) decreased in each treatment group, particularly in the ESTP treatment group (Figure 4c–f). Besides, it is well-known that excessive reactive oxygen species (ROS) has been demonstrated to aggravate the process of VSMCs trans-differentiation into osteoblast-like cells by upregulating RUNX2, and apoptotic VSMCs have the ability to concentrate calcium as nucleating structures for calcium crystal formation, which can promote the development of VC.^38^ Thus, we first examined the levels of ROS in VSMCs with the treatment of STS or EVs-based nanodrugs (EVs, ES, and ESTP) under CM conditions for 48 h. As expected, data from 2,7-dichlorodi-hydrofluorescein diacetate (DCFH-DA) staining revealed that both ES and ESTP had the best effect of ROS reduction on VSMCs as compared with other treatment groups (Figure 4g,h). Additionally, the images of TUNEL staining showed that the apoptosis of mouse VSMCs in each therapy group was significantly lower than that of the model group, and the order of their efficiency of apoptosis reduction was as follows: ESTP > ES > EVs > STS (Figure 4i,j). The similar findings were also observed by flow cytometry using an annexin V-FITC/Propidium Iodide (PI) assay (Figure 4k,l).

### ESTP Nanodrugs Actively Targeted Calcified Vascular Tissue and Exhibited Efficient Therapeutic Efficacy for VC *In Vivo*

**Figure 5:**
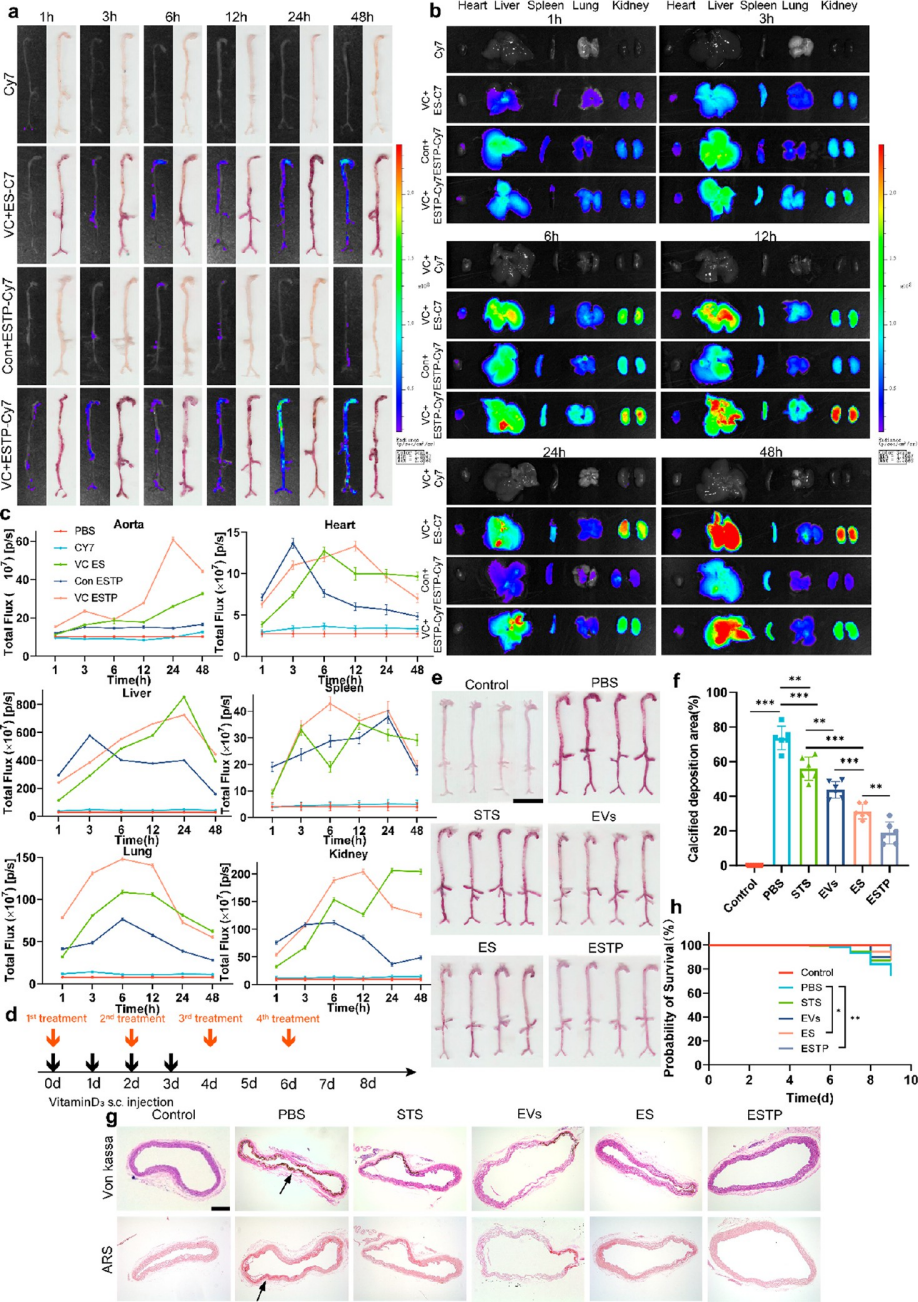
**Targeting efficiency, biodistribution, and therapeutic efficacy of ESTP*****in vivo***. (a, b) Bioluminescence images and the (c) semiquantitative analysis of Cy7 fluorescence of (a) aorta and (b) major organs (heart, liver, spleen, lung, and kidney) collected from mice at different time points from mice with or without VC after intraperitoneal injection of free Cy7, Cy7-labeled ESs, and Cy7-labeled ESTP. (d) Schematic illustration showing the design of animal experiments. (e) Representative Alizarin Red S staining and the (f) quantification of positive areas of calcified aortas (from the ascending aortic root to the iliac bifurcation). Scale bar: 1 cm. (g) Representative staining of von Kossa and Alizarin Red S of aortic arch sections. The black arrows indicate the calcified aortas (*n* = 3). Scale bar: 200 μm. (h) Survival curves of mice with various treatments (*n* = 15). *P* value style: *P < 0.05; **P < 0.01; or ***P < 0.001. Data are presented as mean ± standard deviation (SD).

The *in vivo* biodistribution and VC targeting capacity of free Cy7, Cy7-labeled ES (Cy7-ES), and Cy7-labeled ESTP (Cy7-ESTP) were investigated in control or a VC mice model via intraperitoneal injection. Mice were sacrificed at 1, 3, 6, 12, 24, and 48 h after administration. Aortas and main organs (heart, liver, spleen, lung, and kidney) were then excised in the dark to compare the levels of fluorescence accumulation. Interestingly, the results from aortas showed that the fluorescent intensity of Cy7-ESTP was obviously higher than that of free Cy7 and Cy7-ES at the same time points in the mice VC model. Of note, these positive areas of strong fluorescence of Cy7-ESTP were consistent with the sites of VC which were confirmed by Alizarin Red S staining (Figure 5a,c, Figure S10). However, no obvious fluorescence signaling was observed in mice without VC. Besides, fluorescence intensity of Cy7-ESTP at heart, liver, spleen, lung, and kidney was lower than Cy7-ES in model mice after administration for 24 h (Figure 5b,c, Figure S10). These results suggested that our ESTP nanodrugs had good VC targeting. We next detected STS concentration in mice plasma at different time points after intraperitoneal injection of free STS, ES, and ESTP using high-performance liquid chromatography (HPLC). In comparison, the half-life of STS in ESTP was longer than that in ES and STS, and then STS in ESTP went through a longer elimination phase with a half-life of about 61.7 h (Figure S11a,b). These results indicated that ESTP could prolong circulation time of STS by enhancing VC targeting and reducing the retention into other organs such as liver, spleen, and kidney. Subsequently, the anti-VC efficacy of ESTP on VC mice model were evaluated (Figure 5d). In order to explore the better therapeutic effect and less side effects of ESTP on VC, 50 mg kg^–1^ of STS, an eighth of therapeutic concentration for VC,^13^ was chosen for subsequent animal experiments. Compared with other groups, mice treated with ESTP exhibited an excellent reduction in the area of VC (Figure 5e,f). Also, the anti-VC efficacy of ES was more obvious than monotherapy of EVs or STS, which indicated that EVs and STS had synergistic effects on VC (Figure 5e,f). Similar findings were also observed in Von kassa, Alizarin Red S staining (Figure 5g). In addition, aorta sections were subjected to TUNEL staining to determine the apoptotic level, and the results demonstrated that the apoptotic cells were obviously reduced in VC mice treated with ESTP (Figure S12). In survival analysis, ESTP showed the highest probability of survival compared with other treatment groups (Figure 5h). Next, to evaluate the biosafety and systemic toxicity of ESTP, we assessed other main organs, biochemical indicators, and hemolysis in mice after treatment. Hematoxylin and eosin (H&E) staining of the main organs showed that there was no obvious tissue damage except for the 8STS group. Results showed that treatment with 8STS caused hepatocyte swelling and karyopyknosis in the liver; splenomegaly, infiltrated inflammatory cells, and follicular hyperplasia in the spleen; a widened alveolar septum, alveolar congestion, and pulmonary atelectasis in the lung; the epithelium of renal tubule shed off into the lumen, renal tubular distension, and abundant inflammation cells infiltration in the kidney (Figure S13). Concurrently, serum biochemical indicators related to liver function (alanine aminotransferase (ALT), aspartate aminotransferase (AST)) and kidney functions (creatinine (CREA), urea nitrogen (BUN)) were significantly improved in each treatment group, particularly in the ESTP treatment group (Figure S14). Hemolysis was not observed for EVs, ES, and ESTP at concentrations up to 50 mg·L^–1^ and STS at concentrations up to 20 mM·L^–1^ as compared with water (Figure S15). Taken together, these results indicated the obtained ESTP nanodrugs had good biosafety and without the concern of systemic toxicity *in vivo* and presented excellent capacity to attenuate the progression of VC through enhancing effective accumulation in the site of VC.

### Mechanism of ESTP Nanodrugs in Inhibiting VC

**Figure 6:**
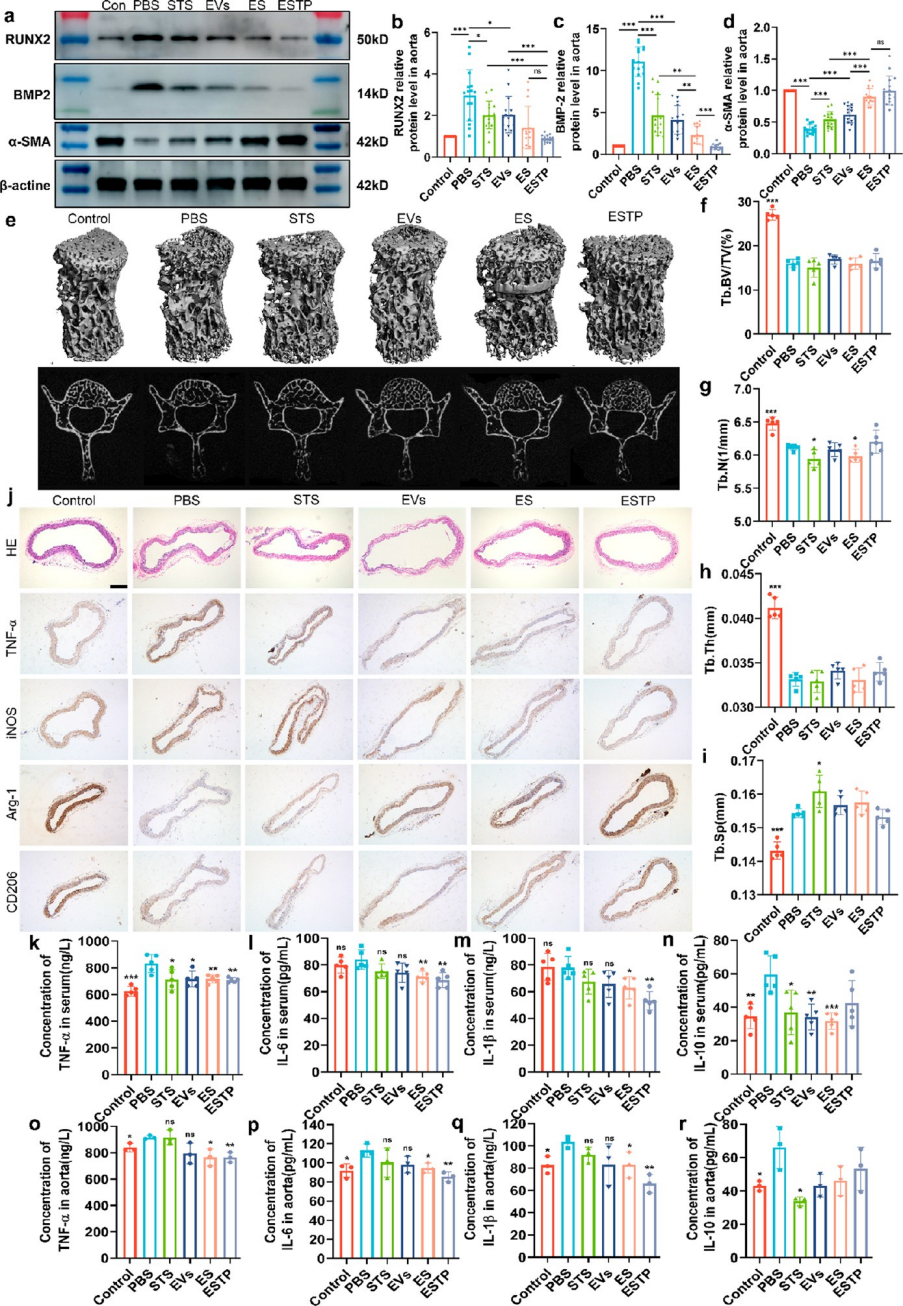
**Inflammation response and bone-vascular axis were suppressed by ESTP**. (a) Representative Western blot analysis and quantification of the (b, c) osteogenic (RUNX2 and BMP2) and (d) contractile (α-SMA) protein expression in aortas of VC mice with PBS, STS, EVs, ES, or ESTP treatment. (e) Micro computed tomography (micro-CT) images of the fifth lumbar vertebra and quantification of (f) trabecular bone volume fraction (Tb. BV/TV), (g) trabecular number (Tb. N), (h) trabecular thickness (Tb. Th), and (i) trabecular separation (Tb. Sp) (*n* = 6). (j) H&E staining of aortic arch sections from each group, and the immunohistochemical staining was performed using anti-TNF-α antibody and anti-iNOS antibody as M1 macrophage marker, anti-Arg-1 antibody and anti-CD206 antibody as M2 macrophage marker (*n* = 3). Scale bar: 200 μm. The serum levels of (k) TNF-α, (l) IL-6, (m) IL-1β, and (n) IL-10 were determined by ELISA (*n* = 5). The levels of (o) TNF-α, (p) IL-6, (q) IL-1β, and (r) IL-10 in aorta homogenates were measured (*n* = 3). *P* value style: *P < 0.05; **P < 0.01; or ***P < 0.001. Data are presented as mean ± standard deviation (SD).

Growing clinical and experimental evidence links bone disorder with the development of VC and demonstrates that bone-vascular axis involved in the pathological process of VC, especially for the osteogenic phenotype differentiation of VSMCs.^39,40^ Thus, we investigated whether ESTP inhibited VC via acting on the bone-vascular axis. Our results from Western blot analysis showed that the expression of contractile phenotype marker (α-SMA) significantly upregulated, whereas the osteogenic phenotype marker (RUNX2 and BMP2) downregulated in each treatment group, particularly in the ESTP treatment group (Figure 6a–d). These results suggested that ESTP was the best strategy to inhibit VSMCs transformation into osteogenic phenotype. Furthermore, previous studies have demonstrated that STS can decrease bone strength and jeopardize bone quality in STS-treated animals (STS of 400 mg kg^–1^),^13^ which is likely by inducing systemic acidosis and/or hypocalcemia. It means that the therapeutic effect of VC is at the expense of systemic side-effects. Herein, the anti-VC effect and side-effects of 50 mg kg^–1^ of STS, 400 mg kg^–1^ of STS (marked as 8STS group), and ESTP were investigated in VC mice model via intraperitoneal injection. Of note, although the concentration of STS in ESTP was an eighth of 8STS, ESTP still showed the best efficacy of anti-VC (Figure S16a). Next, we used microcomputed tomography (micro-CT) analysis to determine the impact of ESTP on bone quality and whether STS-induced osteoporosis could be alleviated by reducing the systemic dose of STS or nanodrugs (EVs, ES, and ESTP). As expected, the results of CT images and trabecular bone microarchitecture parameters of the fifth lumbar vertebras were as follows: (i) Bone density in VC mice model was significantly lower than that in controls, which was due to the disorder of bone-vascular axis and the metabolism disorder of calcium and phosphate.^41^ (ii) VC mice treatment with 8STS caused worse bone quality such as significant low-density area and bone loss, which was consistent with prior studies.^13,14^ (iii) ESTP could effectively rescue trabecular bone destruction induced by STS (Figure 6e–i and Figure S16b–f). Interestingly, our data also showed that EVs significantly reversed bone mechanical strength reduction, which indicated synergistic therapeutic effects of anti-VC with STS in VC mice model. In addition, previous studies have reported that inflammation is the principal common pathway linking the bone-vascular axis.^42^ Specifically, calcium deposition was triggered when monocyte-derived macrophages were recruited and activated, where M1 macrophages powerfully accelerated osteogenic phenotype trans-differentiation of VSMCs via proinflammatory factors.^43^ To fully assess the efficacy of ESTP on regulating the local inflammatory response, aorta sections were subjected to H&E assays to observe inflammatory changes and stained with antibody of M1 macrophages markers (TNF-α and iNOS) and M2 macrophages markers (Arg-1 and CD206). The results demonstrated that the pathology of tissue inflammation in the ESTP group was significantly milder than that in the VC model group and the expression of M1 macrophage markers (TNF-α and iNOS) significantly decreased, whereas the M2 macrophage markers (Arg-1 and CD206) increased in each treatment group, particularly in the ESTP treatment group (Figure 6j). Moreover, ESTP significantly decreased the proinflammatory cytokines (TNF-α, IL-6, and IL-1β) and increased the anti-inflammatory factor IL-10 in both the serums and the aorta tissues (Figure 6k–r). These data suggested that the therapeutic effect of ESTP on VC was mediated by the synergism between EVs and STS of driving M2 macrophage polarization to alleviate inflammation. Collectively, ESTP effectively prevents the progression of VC via driving M2 macrophage polarization, reducing inflammation, and suppressing bone-vascular axis as demonstrated by inhibiting osteogenic phenotype trans-differentiation of VSMCs while enhancing bone quality.

### Therapeutic Targets and Signaling Pathways of Grapefruit-Derived EVs against VC

**Figure 7:**
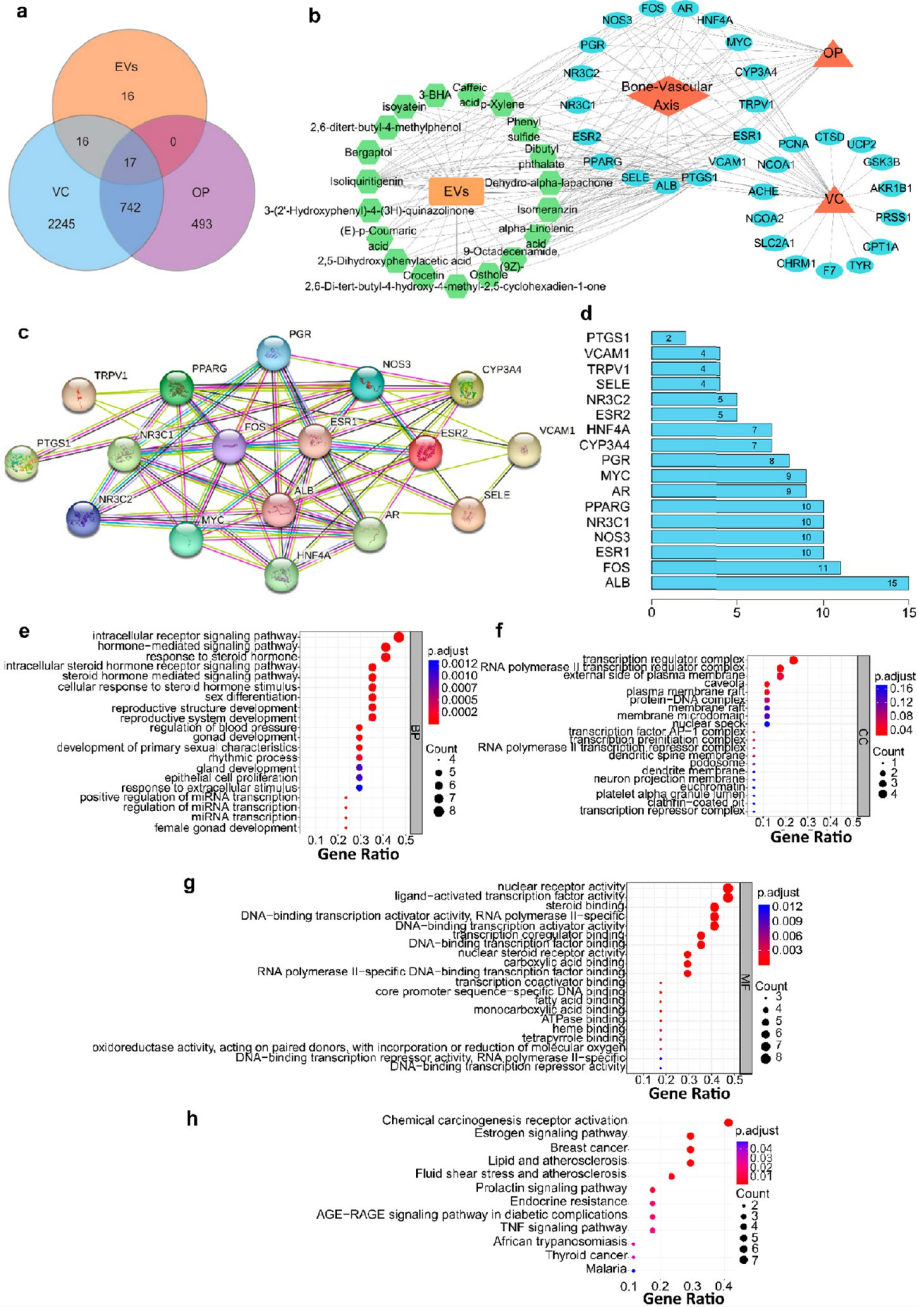
**Therapeutic targets and signaling pathways of grapefruit-derived EVs against bone-vascular axis**. (a) Venn diagram revealing the 17 intersection genes of EVs against VC/OP. (b) The EVs regulatory network in bone-vascular axis. Green hexagons: active components of EVs; Blue ovals: targeting genes. (c) Protein–protein interaction network and (d) statistical analysis of the 17 EVs targets. (e–g) GO and (h) KEGG enrichment analysis of the 17 EVs targets.

To further reveal the pharmacological targets of grapefruit-derived EVs nanodrugs in the bone-vascular axis, we analyzed the chemical components of grapefruit-derived EVs using high-performance liquid chromatography–mass spectroscopy (HPLC-MS) (Figure S17). A total of 27 bioactive components from 2398 identified species were screened out according to content, bioavailability, and drug-likeness using the mzCloud database (https://www.mzcloud.org/) and mzVault database (https://mytracefinder.com/tag/mzvault/) (Tables S1, S2). Next, according to network pharmacology methodology, EVs were regarded as a mixture of chemical ingredients, and EVs-compound-target-disease (VC and osteoporosis (OP)) connections were constructed to explore their potential functional pathways.^44^ Specifically, to reveal the regulatory network of EVs in both VC and OP, a total of 49 pharmacological targets of the 27 bioactive components from EVs were predicted using the TCMSP database (https://old.tcmsp-e.com/tcmsp.php/) and 17 differently expressed common predicted targets of EVs against VC and osteoporosis (OP) were identified by comparing with 2987 VC-associated targets and 965 OP-associated targets using GeneCards (https://www.genecards.org/) and OMIM (https://www.omim.org/) (Figure 7a and Table S3). A component-protein network showed the correlation between disease, drugs, and targets (Figure 7b). It revealed that grapefruit-derived EVs could be effective therapeutic strategies for both VC and OP by regulating shared protein targets of related bone-vascular axis, and these functional characteristics of EVs were multicomponent and multitarget. Besides, the protein–protein interaction network of the 17 intersection targets was analyzed using STRING (https://cn.string-db.org/) (Figure 7c). The results showed that ALB was the hub protein, which is associated with 15 functional proteins (Figure 7d and Table S4). Gene ontology (GO) enrichment analysis indicated that these 17 intersection targets were involved in a series of biological processes, including intracellular receptor regulation and transcription regulation (Figure 7e–g and Table S5). Additionally, Kyoto Enclyclopedia of Genes and Genomes (KEGG) enrichment analysis also indicated that these 17 intersection targets were significantly associated with pathways of receptor activation, estrogen signaling, and atherosclerosis-related signaling (Figure 7h and Table S6). Taken together, these bioinformatic analyses using network pharmacology have highlighted the potential therapeutic targets and pharmacological pathways of grapefruit-derived EVs action against VC.

## Conclusions

We have developed a biomimetic delivery system in which water-soluble and clinically approved drug (STS) was loaded into grapefruit-derived EVs and HA-binding peptide (TP) was modified on the surface of EVs. The obtained ESTP not only produced synergistic therapeutic effects to VC but also significantly reduced systemic toxicity. The distinctive advantages of the ESTP that we report here are listed below. (i) Grapefruit-derived EVs can not only act as delivery carriers but also exert obvious anti-VC function. This discovery can be extended to deliver other potential therapeutic agents to synergistically inhibit VC. (ii) ESTP exhibits excellent cellular uptake capacity by calcified VSMCs *in vitro* and efficient retention in the site of VC *in vivo*. Mechanistically, ESTP can effectively prevent the progression of VC via driving M2 macrophage polarization, reducing inflammation, and suppressing bone-vascular axis as demonstrated by inhibiting osteogenic phenotype trans-differentiation of VSMCs while enhancing bone quality. In summary, this work provides a special delivery system for nanodrugs, which is promising for clinical translation as a treatment for VC without the concern of systemic toxicity.

## Methods/Experimental

### Materials and Reagents

Sodium thisulfate standard solution (STS, S196964) was purchased from Aladdin Biological Technology Co., Ltd. (Shanghai, China). SP5–52-SH peptide, DSPE-(PEG)_2000_-maleimide (R-0039–2k), and Sulfo Cyanine7-NHS(R-H-1709) ester were synthesized by Xi’an ruixi Biological Technology Co., Ltd. (Xi’an, China). Bicinchoninic acid (BCA) protein assay kit (P0012) and Reactive Oxygen Species assay kit (S0033S) were purchased from Beyotime Biotechnology Co., Ltd. (Shanghai, China). The Annexin V-FITC/PI cell apoptosis detection kit (FA101–02) was purchased from TransGen Biotech Co., Ltd. (Beijing, China), and the TUNEL FITC apoptosis detection kit (A111–02) was purchased from Vazyme Biotech Co., Ltd. (Nanjing, China). Alix (ab275377), CD9 (ab223052), CD63 (ab216130), calnexin (ab22595), GAPDH (ab181602), beta Actin (ab8227), BMP2(ab284387), RUNX2(ab23981), alpha smooth muscle actin (ab5694) primary rabbit antibodies, and Goat Anti-Rabbit secondary antibodies (ab7085) were purchased from Abcam company (USA). Sucrose VetecTM reagent grade (V900116) and sodium phosphate dibasic solution (BCCD7444) were purchased from Sigma-Aldrich (USA). Cholecalciferol (D3) (V330571) was purchased from Aladdin (Shanghai, China). PKH26 dye (PKH26GL) was purchased from Sigma-Aldrich (USA). FITC Phalloidin (40735ES75) was purchased from Yeason Biotechnology Co., Ltd. (Shanghai, China). DAPI (C1002) was purchased from Beyotime Biotechnology Co., Ltd. (Shanghai, China). Mouse Interleukin 6 (IL-6) ELISA KIT (69-99854), mouse Interleukin 1β (IL-1β) ELISA KIT (69-21178), mouse Interleukin 10 (IL-10) ELISA KIT (69-99847), mouse tumor necrosis factor-α (TNF-α) ELISA KIT (69-99985), mouse alanine aminotransferase (ALT) ELISA KIT (69-50087), mouse aspartate aminotransferase (AST) ELISA KIT (69-36251), mouse serum creatinine (CREA) ELISA KIT (69-21413), and mouse blood urea nitrogen (BUN) ELISA KIT (69-21234) were purchased from Merck Biotechnology Co., Ltd. (Wuhan, China).

### Grapefruit-Derived EVs Isolation and Purification

For isolation of grapefruit-derived EVs, grapefruits (Genus, Citrus L.; Family, Rutaceae; Order, Rutales; Subclass, Archichlamydeae; Class, Dicotyledoneae) were purchased from farmer’s markets. Grapefruits were washed three times, skin removed, and squeezed juice. Next, the juice was differentially centrifuged at 500*g* for 10 min, 2000*g* for 20 min, 5000*g* for 30 min, and 10,000*g* for 1 h to remove the residues and fibers of grapefruits. And then, the supernatant was ultracentrifuged at 100,000*g* for 2 h, and sedimentation was then resuspended in PBS.^45^ Finally, to purify the EVs, the suspension was ultracentrifuged on a sucrose gradient (8, 30, 45, and 60% (w/v) sucrose in 20 mM Tris. HCl, pH 7.2) at 150,000*g* for 2 h, and the band from the interface of 30%/45% was then harvested and noted as EVs.^45^ The concentration of EVs was quantified by BCA protein assay kit; membrane proteins of EVs were identified by Western blot analysis; morphology of EVs was observed by transmission electron microscopy (TEM), and size distribution and Zeta potential of EVs were measured by dynamic light scattering (DLS) using a Zetesizer Nano ZS.^46^

### HA-Binding Peptide Synthesis

HA-binding peptide (SP5–52-SH) was synthesized as previously described.^9^ In brief, HA-binding peptide of sequence [CSVSVGMKPSPRP] was synthesized using standard Fmoc-mediated solid-phase peptide synthesis on an automatic PS3 benchtop peptide synthesizer (Ruixi Biological Technology Co., Ltd., Xi’an, China). The cysteine residue at the N-terminus of the peptides was used for the thioether linkage. The peptides were then N-capped with an acetyl group to obtain free *hydrosulphonyl* (-SH) at the N-terminus and free amino of side chain which can bind with calcium ion and phosphate ion in HA.^9^ The peptides were cleaved from the rink amide resin in the cutting solution (94:2.5:2.5:1 volume ratios of trifluoroacetic acid/1,2 ethanedithiol/H_2_O/triisopropylsilane). Then, the peptides were chromatographed out with ether and washed six times with ether. When the peptides were dried at room temperature, the crude peptide sequence was obtained. Next, the crude peptides were purified by a high-performance liquid chromatography (HPLC) system with initial gradient using 95:5 volume ratios of water/acetonitrile and the terminal gradient using 75:25 volume ratios of water/acetonitrile, and the flow time was 40 min. Purified samples were further verified by using mass spectral analysis.

### Preparation of ES

For preparation of ES, EVs and an equal volume sodium thiosulfate solution (STS, S196964, Aladdin Biological Technology Co., Ltd., Shanghai, China) with different concentrations were added in PBS, and the mixtures were then reacted at room temperature for 24 h. Subsequently, the uncombined STS was removed by ultracentrifugation at 100,000*g* for 1 h, and the ES was then washed once using ultracentrifuge at 100,000*g* for 30 min. To quantify the loading efficiency of STS in ES, the contents of uncombined STS in supernatant were measured by UV spectrophotometer at the wavelength of 215 nm according to the standard calibration curves of STS. The loading efficiency of STS in ES was calculated by the following equation.
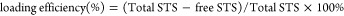
1The loading weight of STS in 1 mg of EVs was calculated by the following equation.
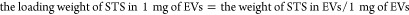
2

### Preparation of ESTP

First, pure SP5–52-SH peptide (TP) and equimolar DSPE-(PEG)_2000_-MAL (R-0039–2k, Xi’an ruixi Biological Technology Co., Ltd., Xi’an, China) were added into PBS (PH 7.4) at 4 °C for 24 h to synthesize DSPE-(PEG)_2000_-TP by *sulfydryl* (-SH) and - *maleimide* (MAL) addition reactions.^34^ Successful synthesis of DSPE-(PEG)_2000_-TP was confirmed by sodium dodecyl sulfate/polyacrylamide gel electrophoresis (SDS-PAGE). Next, ES and DSPE-(PEG)_2000_-TP with the range of feed ratio TP/ES(m/m) from 0:1 to 8:1 were added in PBS, and the mixture was then stirred at 4 °C for 3 h. Finally, unconjugated DSPE-(PEG)_2000_-TP was removed by ultracentrifugation at 100,000*g* for 1 h, and the ESTP was then washed once using ultracentrifuge at 100,000*g* for 30 min. In order to quantify the combined efficiency of DSPE-(PEG)_2000_-TP in ESTP, the amount of free DSPE-(PEG)_2000_-TP in the supernatant was measured by a BCA protein assay kit according to the manufacturer’s instruction. The combined efficiency was calculated as follows.
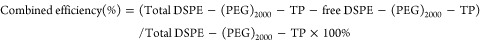
3To determine the optimal ratio of ES/TP in ESTP, the size distribution and Zeta potential of ESTP were measured by DLS using a Zetesizer Nano ZS. At the same time, we observed effects of different products on mouse VSMCs calcification models.

### Stability of EVs, ES, and ESTP

EVs, ES, and ESTP were suspended in 10% FBS for 168 h at 37 °C to evaluate stability in simulated *in vivo* condition. Further, EVs, ES, and ESTP were suspended in PBS (PH 7.4) and stored for 30 days at 4 °C to evaluate stability under storage condition. The changes of size distribution and Zeta potential of nanodrugs were used to evaluate stability. Data are Mean ± standard error of measurement (SEM). All experiments were independently performed at least three times.^47^

### *In Vitro* STS Release Profile from ES and ESTP

To test the *in vitro* release of STS from ES and ESTP, 1 mg of STS, ES, and ESTP (containing 1 mg of STS) were dissolved in 1 mL of PBS (PH 7.4), and the solutions were then sealed in the dialysis membranes (MD10, molecular weight cutoff (MWCO): 1000, USA). Subsequently, the devices were immersed in 100 mL of incubation media (PBS) at 37 °C with shaking slowly. At the predetermined time points (0, 1, 2, 4, 8, 16, 24, and 48 h), 0.5 mL of incubation media was taken out to analyze and 0.5 mL of fresh PBS was replenished into the incubation media. STS concentrations were determined by UV–vis spectrometer (215 nm) according to standard curves in PBS.^45^ The cumulative release rate of STS was calculated as follows
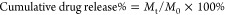
4where *M*_t_ was the concentration of STS in incubation media at time *t* and *M*_0_ was the initial concentration of STS in incubation media.

### Western Blotting

Cells, tissues, EVs, ES, and ESTP were lysed with radioimmunoprecipitation assay (RIPA) lysis buffer (P0013B, Beyotime Biotechnology, Shanghai, China) supplemented with 1% protease and phosphatase inhibitor (78440, Thermo Fisher Scientific, USA) to extract total proteins. The concentration of the total protein was measured using a BCA protein assay kit. A total of 50 μg of protein lysates were separated by 8–12.5% sodium dodecyl sulfate/polyacrylamide gel electrophoresis (SDS/PAGE) and then transferred on to polyvinylidene fluoride (PVDF) membranes (Millipore, USA). The PVDF membranes were then blocked for 2 h at room temperature and incubated with primary antibodies for 13 h at 4 °C as follows: rabbit anti Alix antibody (1:1000, ab275377, Abcam, USA), rabbit anti CD9 antibody (1:1000, ab223052, Abcam, USA), rabbit anti CD63 antibody (1:1000, ab216130, Abcam, USA), rabbit anti BMP2 antibody (1:1000, ab284387, Abcam, USA), rabbit anti RUNX2 antibody (1:1000, ab23981, Abcam, USA), and rabbit anti α-SMA antibody (1:1000, ab5694, Abcam, USA). Next, the PVDF membranes were washed three times with Tri-*sec*-Buffered Saline Tween (TBST) for 10 min and were then incubated with Goat Anti-Rabbit secondary antibody (dilution 1:10000) for 2 h. Finally, the signals were visualized using an Imaging System (GE, Amersham Imager 600, Piscataway, NJ, USA). The gray scale values of the target protein band were quantified using ImageJ software.

### Cell Culture and *In Vitro* Calcification Model

Primary mice VSMCs were isolated from thoracic aortas of 4-week-old male C57BL/6 mice as previously described^48^ and maintained in the Dulbecco’s modified Eagle’s medium (DMEM, C11995500BT, Gibco, USA) supplemented with 10% FBS (1099–141, Gibco, USA), 100U mL^–1^ penicillin, and 100 mg mL^–1^ streptomycin (Thermo Fisher, USA) at 37 °C in a humidified atmosphere containing 5% CO_2_. The cells at passages 3 to 8 were used for the *in vitro* experiments. Each experiment was repeated at least three times. VSMCs calcification was induced by calcifying medium (CM) containing 3 mM sodium phosphate dibasic solution (BCCD7444, Sigma-Aldrich, USA) for 7 days with medium changes every 2 days.

### Cytotoxicity Assay *In Vitro*

Mouse VSMCs were seeded in a 96-well plate at a density of 1 × 10^4^ /well and incubated overnight. Subsequently, each well was washed once with PBS to remove dead cells and then replenished with fresh medium containing different concentrations of STS, EVs, ES, and ESTP. After incubation for 48 h, medium was removed and cells were thoroughly rinsed once with PBS. Cells were then incubated with 100 μL/well of fresh medium containing 10% CCK8 solution at 37 °C. At the time points (0.5, 1, 2, and 4 h), the absorbance was measured at 450 nm on a microplate reader (Thermo Scientific, USA). Cell viability in each group was calculated as follows.
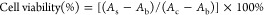
5Here, *A*_s_ was the absorbance of experience group, *A*_b_ was the absorbance of blank control group, and *A*_c_ was the absorbance of control group.

### Intracellular Localization and Cellular Uptake Assay

For imaging of cellular uptake of EVs, ES, and ESTP *in vitro*, mouse VSMCs with the concentration of 4 × 10^4^ per well were seeded in confocal dishes (801002, NEST, Wuxi, China) and cultured overnight. The culture medium was replaced with 1 mL of general medium (GM) or CM and incubated for 48 h. Then, the culture medium of each well was changed with 1 mL of fresh medium containing PKH26-labeled EVs, ES, and ESTP (5 mg L^–1^ EVs, red channel) and incubated at 37 °C. At the predetermined time points (1, 3, 6, 12, and 24 h), cells were fixed with 4% paraformaldehyde for 30 min and then labeled with phalloidin-FITC (green channel) and 4,6-diamidino-2–2-phenylindole (DAPI) (blue channel) to display the distribution of F-actin and nucleus, respectively. Finally, cells were observed and imaged using a fluorescence microscope (DM4000B, Leica, Germany) with Leica Application Suite X software version 3.7.4.23463. Mouse VSMCs were seeded in six-well plates at an initial density of 1 × 10^6^ per well and incubated overnight. Flow cytometry was used for quantification of cellular uptake. Methods of culture, inducing calcification and intervention were as described previously. At the predetermined time points (1, 3, 6, 12, and 24 h), cells were thoroughly washed three times with cold PBS and then centrifuged at 800 r min^–1^ for 3 min. Next, cells were resuspended in 1 mL of PBS solution for detection of PKH26 signal (PE channel) using flow cytometry (Backman, USA). The results were analyzed by FlowJo 10 software. All experiments were independently performed at least three times.

### Efficacy for Calcification Models In Vitro

To investigate the effects of different treatments on VC *in vitro*, mouse VSMCs were seeded in 6-well plates at a density of 1 × 10^6^ per well and incubated overnight. Subsequently, the medium was changed on alternate days until the cell density reached up to 60–70%. Next, the culture medium was replaced with 2 mL of GM or CM. At the same time, STS (1 mM), EVs (5 mg L^–1^), ES, and ESTP (the concentration STS of 1 mM and EVs of 5 mg L^–1^) were supplemented into the presence of CM to intervene cells for 3 to 7 days. The protein expressions of RUNX2, BMP2, and α-SMA in cells were analyzed by Western blotting at day 4. Calcium deposition in cells was detected by Alizarin Red S staining on day 7. All experiments were independently performed at least four times.

### Intracellular ROS Level Detection

For detection of the level of intracellular reactive oxygen species (ROS), mouse VSMCs were seeded in 6-well plates at a density of 1 × 10^6^ per well and incubated overnight. Subsequently, the culture medium was changed on alternate days until the cell density reached up to 60–70%. Next, STS (1 mM), EVs (5 mg L^–1^), ES, and ESTP (the concentration STS of 1 mM and EVs of 5 mg L^–1^) were then supplemented into the presence of CM to intervene mouse VSMCs for 48 hours. Finally, cells were washed twice with PBS before 2,7-dichlorodi-hydrofluorescein diacetate (DCFH-DA, S0033S, Beyotime, China) staining. Green fluorescence of dichlorofluorescein (DCF) was generated by the reaction of DCFH-DA and intracellular ROS. Cells were observed and imaged using a fluorescence microscope (DM4000B, Leica, Germany) with Leica Application Suite X software version 3.7.

### Cell Apoptosis

For observation of the effects of different treatments on cell apoptosis *in vitro*, mouse VSMCs with the concentration of 4 × 10^4^ per well were seeded in confocal dishes (801002, NEST, Wuxi, China) and cultured overnight. Subsequently, the culture medium was replaced with 1 mL of GM or CM and incubated for 48 h; STS (1 mM), EVs (5 mg L^–1^), ES, and ESTP (the concentration STS of 1 mM and EVs of 5 mg L^–1^) were then supplemented into the presence of CM to intervene cells at 37 °C for 48 h. Finally, cells were fixed with 4% paraformaldehyde for 30 min, and then labeled with TUNEL FITC apoptosis detection kit (green channel, A111–02, Vazyme, Nanjing, China) and 4,6-diamidino-2–2-phenylindole (DAPI) (blue channel) to display the terminal dUTP nick end of DNA breakage and nucleus, respectively. Cells were observed and imaged using a fluorescence microscope (DM4000B, Leica, Germany) with Leica Application Suite X software version 3.7. The Annexin V-FITC/PI cell apoptosis detection kit (FA101–02, TransGen, Beijing, China) was used to quantify cell apoptosis *in vitro*. After the end of incubation, mouse VSMCs were digested by trypsin, washed twice with cold PBS, and then suspended in annexin V binding buffer in 1.5 mL centrifuge tubes. Next, 5 μL of annexin V-FITC and equivalent PI were added into tubes and then incubated for 15 min in the dark at room temperature. Meanwhile, cell suspensions of single staining of FITC or PI and unstained were also prepared as comparation. Finally, fluorescence signals in cell suspensions were detected by annexin V-FITC (FITC channel) signal and PI signal (PE channel) using flow cytometry (Backman, USA). The results were analyzed by FlowJo 10 software. All experiments were independently performed at least three times.

### Animals and In Vivo Treatment

All animal experiments were performed in compliance with the relevant laws and were approved by the Institutional Animal Care and Use Committee at Southern Medical University (Guangzhou, China). Adult male C57BL/6 mice aged 6–8 weeks (16–20 g) were purchased from the Guangdong Medical Laboratory Animal Center. In order to explore the effects of STS, EVs, ES, and ESTP on VC *in vivo*, mice were induced with aortic calcification via subcutaneous injections of cholecalciferol (D3) (20 mg kg^–1^, V330571, Aladdin, Shanghai, China) for four consecutive days. The first day of injection was marked day 0. At the same time, mice were randomly divided into five groups (n = 6), and we intraperitoneally injected the mice with various formulations on alternate days from day 0 to day 8 as follows: (1) VC group, injection of equivalent PBS. (2) STS group, injection of 50 mg kg^–1^ STS (an eighth of therapeutic concentration for VC as previous studies). (3) EVs group, injection of 1.58 mg kg^–1^ of EVs. (4) ES group, injection of ES (50 mg kg^–1^ of STS, 1.58 mg kg^–1^ of EVs). (5) ESTP group, injection of ESTP (50 mg kg^–1^ of STS, 1.58 mg kg^–1^ of EVs). Meanwhile, the control group was intraperitoneally injected with PBS as comparation. At the end of the experiment, mice were sacrificed, and aortas (from the ascending aortic root to the iliac bifurcation) and main organs were then harvested for further analysis.

### In Vivo Biodistribution

For the targeting ability and biodistribution of ESTP in aortic calcification mice, mice with or without aortic calcification were injected intraperitoneally with PBS, free Cy7, Cy7-labeled ES, and Cy7-labeled ESTP with Cy7 concentration 0.02 mg kg^–1^. The amount of Cy7 in ES and ESTP was measured by microplate reader at excitation/emission wavelength: 750/773 nm. It was measured that 1 μg of EVs contained about 0.032 and 0.028 μg of Cy7 in Cy7-labeled ES and Cy7-labeled ESTP. The doses of ES and ESTP in terms of EVs amount administered into the mice were 1.57 and 1.80 mg kg^–1^, respectively (the dose of ES and ESTP was based on therapeutic dose and equivalent Cy7). At the predetermined time points (1, 3, 6, 12, 24, and 48 h) after injection, mice were sacrificed by intraperitoneal injection of 1.5% sodium pentobarbital (5 mL kg^–1^). The aortas (from the ascending aortic root to the iliac bifurcation) and main organs including heart, liver, spleen, lung, and kidney were harvested in the dark and washed by cold PBS to remove surface blood. Finally, the relative accumulation was visualized and compared using an *in vivo* imaging system (IVIS Lumina II, Caliper, USA).^45^

### Pharmacokinetics Analysis In Vivo

C57BL/6 mice were induced with aortic calcification via subcutaneous injections of cholecalciferol (D3) for four consecutive days. The first day of injection was marked as 0 day. On the sixth day, mice were randomly divided into three groups, and STS, ES, and ESTP (50 mg kg^–1^ STS) were then administrated intraperitoneally. At the predetermined time points (0.5, 1, 2, 4, 6, 8, 12, 24, 36, and 48 h) after injection, 100 μL of blood samples was collected from the orbital venous plexus into heparin tubes and then centrifuged at 3000 rpm, 4 °C for 15 min to harvest plasma samples. The amount of STS in plasma was quantified by high-performance liquid chromatography (HPLC) with a UV detector. LC separations were carried out on an XAqua C18 column (4.6 mm × 250 mm, 5 μm, Acchrom, China); the mobile phase consisted of 0.085% (m/v) tetrabutylammonium hydrogen sulfate-water as eluent A and acetonitrile as eluent B, the flow rate was 1.0 mL min^–1^, the column temperature was maintained at 30 °C, the injection volume was 20 μL, and the detection wavelength was 215 nm. The contents of STS were calculated by comparing the absorbance with a standard curve, which was created by adding known contents of STS into mouse plasma. The pharmacokinetic results were analyzed by DAS 2.0 software (China).

### Histopathological Evaluation

For assessment of biosafety *in vivo*, the major organs including heart, liver, spleen, lung, and kidney were harvested and fixed in 4% paraformaldehyde solution for 48 h at room temperature. Subsequently, organs were embedded in paraffin wax and then cut into paraffin sections (5 μm thick). Finally, the tissue sections were stained with hematoxylin and eosin (H&E). To evaluate the effects of different treatments on calcification *in vivo* by pathological staining, the sections of aortic arches were stained with Alizarin Red S, Von kassa, and H&E. For immunohistochemical (IHC) analysis, citrate buffer (pH 6.0) was used for antigen retrieval, and endogenous peroxidase was blocked by 3% hydrogen peroxide solution. Then, the tissue sections were incubated using antibodies against TUNEL, TNF-α, iNOS, Arg-1, and CD206 and detected by 3,3′-diaminobenzidine (DAB) working solution, which results in brown color staining.^49^ The tissue sections were observed and imaged using an optical microscope (DM4000B, Leica, Germany) with Leica Application Suite X software version 3.7. Statistical analysis was performed via ImageJ Pro Plus 6.0.

### Alizarin Red S Staining Analysis

Calcification of mouse VSMCs and aortas was visualized by Alizarin Red S staining. The cells were washed three times with PBS and fixed with 4% paraformaldehyde for 10 min after incubation. Next, cells were washed three times with PBS and then stained by 2% Alizarin Red S (pH 4.2, G8550, Solarbio, Beijing, China) for 5 min. Finally, the cells were washed with deionized water to remove uncombined dye and then imaged using an optical microscope (DM4000B, Leica, Germany) with Leica Application Suite X software version 3.7. To visualize calcification of aortic tissues, the aortas were bluntly isolated to remove the external connective tissue and then fixed with 4% paraformaldehyde for 24 h. Besides, the aortas were incubated overnight with 0.004% (m/v) Alizarin Red S dissolved in 1% KOH in dark at room temperature and then washed with 2% (m/v) KOH for 5 min.^50^ Positively stained samples displayed a red color. Statistical analysis was performed via ImageJ Pro Plus 6.0.

### ELISA Analysis

To quantify the effects of different treatments on inflammatory cytokines and biochemical indicators. After the end of therapy, the mice were sacrificed. Blood samples were collected from the orbital venous plexus and then centrifuged at 3000 rpm and 4 °C for 15 min after clotting for 30 min at room temperature to harvest serum samples. At the same time, the aortas (from the ascending aortic root to the iliac bifurcation) of mice were harvested. Next, aortic tissues with PBS (10 μL mg^–1^ tissue) were homogenized at 60 Hz for 2 min and then centrifuged at 3000 rpm, 4 °C for 15 min to obtain tissue homogenates. Finally, the levels of interleukin 6 (IL-6), interleukin 1β (IL-1β), interleukin 10 (IL-10), tumor necrosis factor-α (TNF-α), alanine aminotransferase (ALT), aspartate aminotransferase (AST), serum creatinine (CREA), and blood urea nitrogen (BUN) in serum samples or tissue homogenates were detected by ELISA KIT (Merck, Wuhan, China) according to the instruction manual.

### Micro-CT Analysis

The vertebral columns (from the thoracic vertebra to the coccyx) of mice were collected and fixed in 4% paraformaldehyde for 2 days. The fifth lumbar vertebras were then subjected to CT scan (caliber, 9 mm; voltage, 55 kVp; current, 200 μA; exposure time, 240 ms; resolution ratio, 10 μm) by micro-CT (SCANCO, Switzerland). The region of interest included the whole volume of cancellous bone within the center of the fifth lumbar vertebra. Images of the region of interest were reconstructed three-dimensionally, and the cancellous bone within centrum of the fifth lumbar vertebra was analyzed by micro-CT Evaluation Program V6.6 to measure trabecular bone volume fraction (Tb. BV/TV), trabecular thickness (Tb. Th), trabecular number (Tb. N), and trabecular separation (Tb. Sp) in different groups.^51^

### Hemolysis Assay

To investigate the hemocompatibility of STS, EVs, ES, and ESTP *in vitro*, a hemolysis test was performed. Whole-blood was collected from healthy C57BL/6 mice and washed five times with PBS to remove leucocyte. Subsequently, red blood cells were incubated with various concentrations STS, EVs, ES, and ESTP for 4 h at 37 °C and then centrifuged at 3000 rpm for 5 min. Finally, the samples were photographed, and the absorbance of supernatants was measured at 576 nm on a microplate reader (Thermo Scientific, USA). Red blood cells were incubated with either ultrapure water or PBS to use as a positive (+) control or negative (−), respectively. The hemolytic ratio was calculated by the following equation.
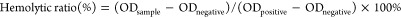
6

### Analysis of Compounds in EVs by HPLC-MS

To analyze compounds in EVs, the sample preparing method was as follows: 1 mg of EVs was ultracentrifuged at 150,000*g* for 1 h and extracted with 1 mL of methanol in an ultrasonic condition for 30 min. Next, the extracts were centrifuged at 14,000 rpm for 20 min, and the supernatant was then filtered through a 0.22 μm membrane filter and diluted by adding different volumes of methanol for HPLC-MS analysis. HPLC analysis was implemented using a Vanquish Ultraperformance Liquid Chromatography System (Thermo Scientific, Germering, Germany) and controlled by the Xcalibur 3.0 software (Thermo Scientific, MA, USA). Chromatographic separations were achieved using a Hypersil GOLD C18 Selectivity HPLC Column (100 mm × 2.1 mm, 1.9 μm; Thermo Scientific, USA). The mobile phase was 0.1% aqueous formic acid–water (A) and methanol (B), the column temperature was maintained at 30 °C, the injection volume was 2 μL, and the gradient was held at 80% B for 0–15 min. MS analysis was acquired using Thermal Oribitrap Fusion Lumos (Thermo Scientific, MA, USA) with a heating electrospray interface (H-ESI). The main parameters included the positive ion spray voltage of 3800 V and the negative ion spray voltage of 2700 V; sheath gas, 40Arb; aux gas, 10Arb; sweep gas, 1Arb; ion transfer tube temperature, 320 °C and vaporizer temperature, 300 °C. Mass spectra were recorded in the *m*/*z* 100–1000 range with accurate mass measurements of all mass peaks.^52^

### Statistical Analysis

All data are presented as the mean ± the standard deviation (SD). Student’s *t* test (two-tailed) was used for comparisons of two groups comparisons. One-way analysis of variance (ANOVA) was used for multiple comparisons. For all statistical tests, two-tailed *P*-value <0.05 indicated statistical significance.

## Data Availability

Supporting Information files are available from the author upon reasonable request.

## Supporting Information Available

Figure S1: SDS-PAGE analysis of DSPE-(PEG)_2000_-TP. Figure S2: Quantitative detection of STS by UV spectrophotometer. Figure S3: The characterization of ESTP in different feeding ratios of TP/ES(m/m). Figure S4–S5: Effects of ESTP and SP5–52-SH peptide (TP) on calcification of mouse VSMCs. Figure S6: The stability of nanoparticles under storage condition. Figure S7: The cytotoxicity evaluation of STS. Figure S8: The cellular uptake of nanoparticles. Figure S9: Effects of STS and EVs on calcification of mouse VSMCs. Figure S10: The biodistribution of Cy7. Figure S11: Pharmacokinetic analysis of nanoparticles. Figure S12: TUNEL staining of aortic arch sections of nanoparticles-treated mice. Figures S13–S15: In vivo safety evaluation of different treatments. Figure S16: Evaluation of anti-VC effect and bone quality in nanoparticles-treated mice. Figure S17: HPLC-MS analysis of the compounds from EVs. Table S1: A total of 2398 chemical components of grapefruit-derived EVs were identified by HPLC-MS. Table S2: A total of 27 bioactive components from grapefruit-derived EVs were screened out according to content, bioavailability and drug-likeness. Table S3: Targets and target symbols corresponding to bioactive compounds. Table S4: Interactions of common predicted targets of grapefruit-derived EVs against VC. Table S5: Main biological processes of grapefruit-derived EVs against VC. Table S6. Major regulatory pathways of grapefruit-derived EVs against VC (PDF)
A total of 2398 chemical components of grapefruit-derived EVs were identified by HPLC-MS (XLS)
A total of 27 bioactive components from grapefruit-derived EVs were screened out according to content, bioavailability, and drug-likeness (XLS)
Targets and target symbols corresponding to bioactive compounds (XLS)
Interactions of common predicted targets of grapefruit-derived EVs against VC (XLS)
Main biological processes of grapefruit-derived EVs against VC (XLS)
Major regulatory pathways of grapefruit-derived EVs against VC (XLS)

## Abbreviations

VC: vascular calcification
EVs: extracellular vesicles
HA: hydroxyapatite crystals
STS: sodium thiosulfate
VSMCs: vascular smooth muscle cells
TP: targeting peptide
CT: computed tomography
TEM: transmission electron microscopy
ROS: reactive oxygen species
ALT: alanine aminotransferase
AST: aspartate aminotransferase
CREA: creatinine
BUN: urea nitrogen
Tb. BV/TV: trabecular bone volume fraction
Tb. N: trabecular number
Tb. Th: trabecular thickness
Tb. Sp: trabecular separation
HPLC-MS: high performance liquid chromatography–mass spectroscopy
OP: osteoporosis
