# Supplementary material for: Biomimetic Grapefruit-Derived Extracellular Vesicles for Safe and Targeted Delivery of Sodium Thiosulfate against Vascular Calcification
Source: ACS Nano. 2023 Dec 6;17(24):24773–89. doi: 10.1021/acsnano.3c05261 (PMC10753875; doi:10.1021/acsnano.3c05261)
Supplement: Supplementary file 1 — nn3c05261_si_001.pdf [file nn3c05261_si_001.pdf]

Supporting Information

Bioinspired Grapefruit-Derived Extracellular Vesicles Integrated Sodium Thiosulfate for Vascular Calcification Treatment

Weijing Feng[1,2‡], Yintong Teng[1‡], Qingping Zhong[1], Yangmei Zhang[1], Jianwu Zhang[2], Peng Zhao[3], Guoqing Chen[4], Chunming Wang[5], Xing-Jie Liang[6], Caiwen Ou[1*]*

[a]The Tenth Affiliated Hospital of Southern Medical University (Dongguan People's Hospital), Southern Medical University or The First School of Clinical Medicine, Southern Medical University, Dongguan 523018, China.

[b]Department of Cardiology, State Key Laboratory of Organ Failure Research, Guangdong Provincial Key Laboratory of Cardiac Function and Microcirculation, Nanfang Hospital, Southern Medical University, Guangzhou, 510515, China

[c]NMPA Key Laboratory for Research and Evaluation of Drug Metabolism, Guangdong Provincial Key Laboratory of New Drug Screening, Guangdong Provincial Key Laboratory of Cardiac Function and Microcirculation, School of Pharmaceutical Sciences, Southern Medical University, Guangzhou 510515, China

[d]Cardiology Department of Panyu Central Hospital and Cardiovascular Disease Institute of Panyu District, Guangzhou 511400, China

[e]Institute of Chinese Medical Sciences & State Key Laboratory of Quality Research in Chinese Medicine, University of Macau, Macau SAR, China

[f]Chinese Academy of Sciences (CAS) Center for Excellence in Nanoscience and CAS Key Laboratory for Biomedical Effects of Nanomaterials and Nanosafety, National Center for Nanoscience and Technology, Beijing 100190, China

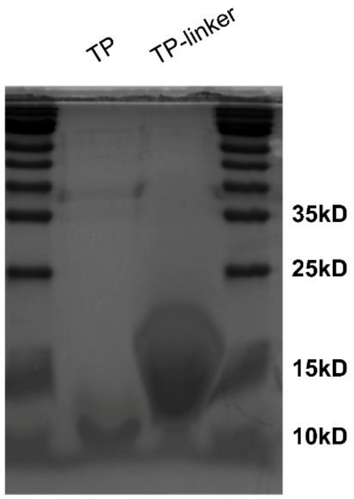

Figure S1. SDS-PAGE analysis. Left to right: Ladders, SP5-52(TP) (30μg), DSPE-$(PEG)_{2000}$-TP (30μgTP), Ladders.

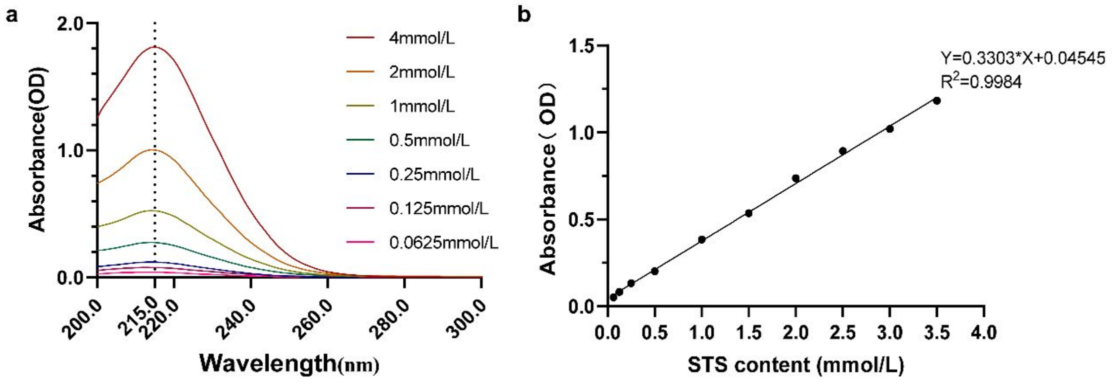

Figure S2. Quantitative detection of STS by UV spectrophotometer. a) UV-vis spectra of STS. b) Standard curve of STS at the wavelength of 215 nm.

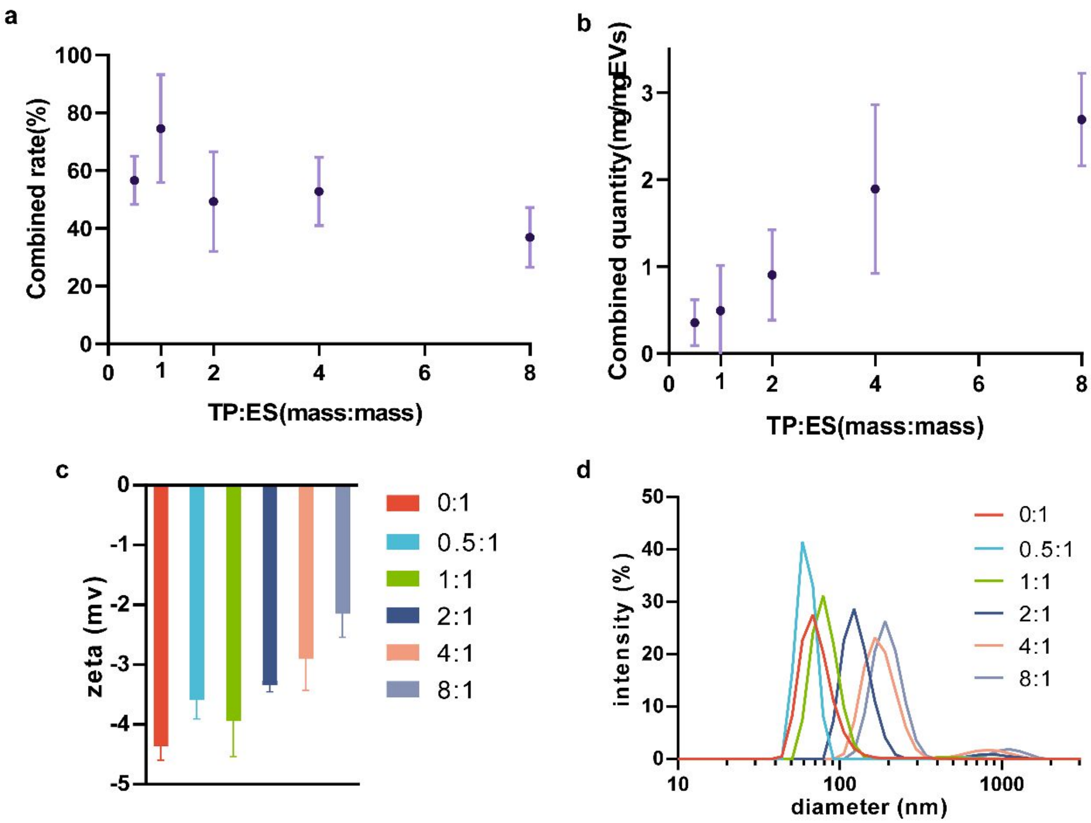

Figure S3. The characterized property of ESTP with the range of feed ratio TP/ES(m/m) from 0:1 to 8:1. a) Combined rate and b) combined quantity of TP with ES; c) Zeta potentials and d) size distribution of ESTP (n=5). Data were presented as means ± SD.

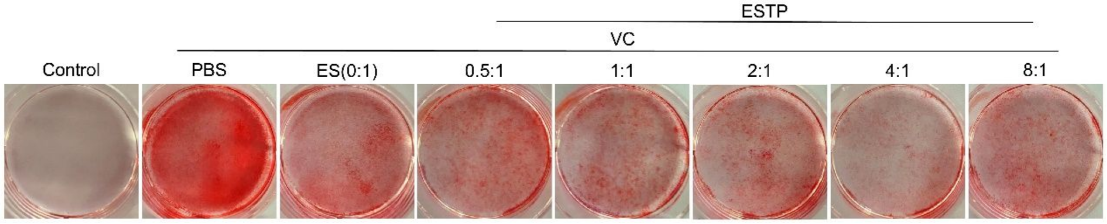

Figure S4. Effect of ESTP on calcification of mouse VSMCs. Mouse VSMCs were incubated with ESTP with the range of feed ratio TP/ES(m/m) from 0:1 to 8:1 (5 mg L^{-1} EVs) in the calcifying medium for 7 days. Mineral deposition in mouse VSMCs was detected by alizarin red staining (n=3).

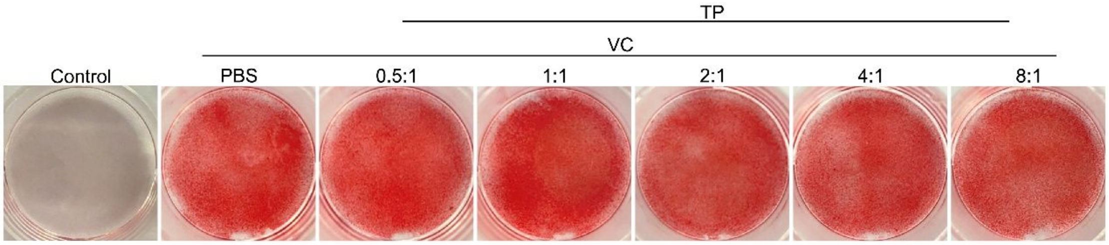

Figure S5. Effect of SP5-52-SH peptide (TP) on calcification of mouse VSMCs. Mouse VSMCs were incubated with free TP (the concentration is equivalent to ESTP) in the calcifying medium for 7 days. Mineral deposition in mouse VSMCs was detected by alizarin red staining (n=3).

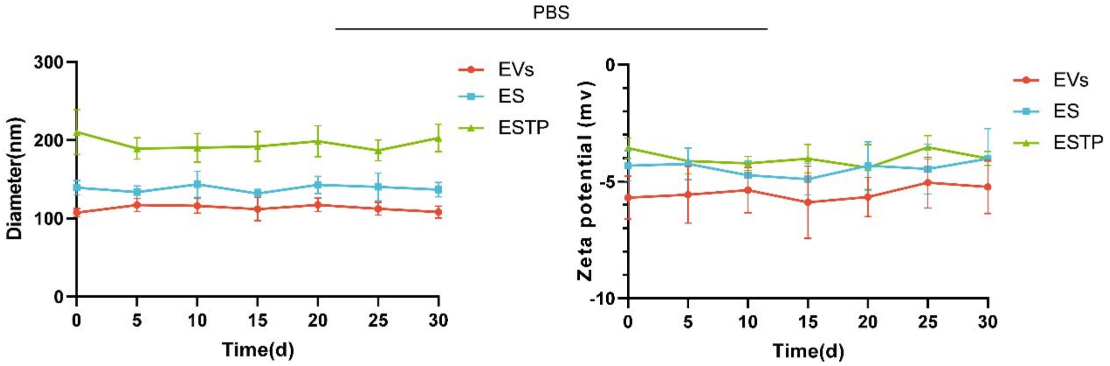

Figure S6. Evaluation of stability of nanoparticles under storage condition. EVs, ES and ESTP were suspended in PBS (pH=7.4) and stored at 4° C for 30 days. The stability of EVs, ES and ESTP was indicated by changes of size distribution and Zeta potential (n=3). Data were presented as means ± SD.

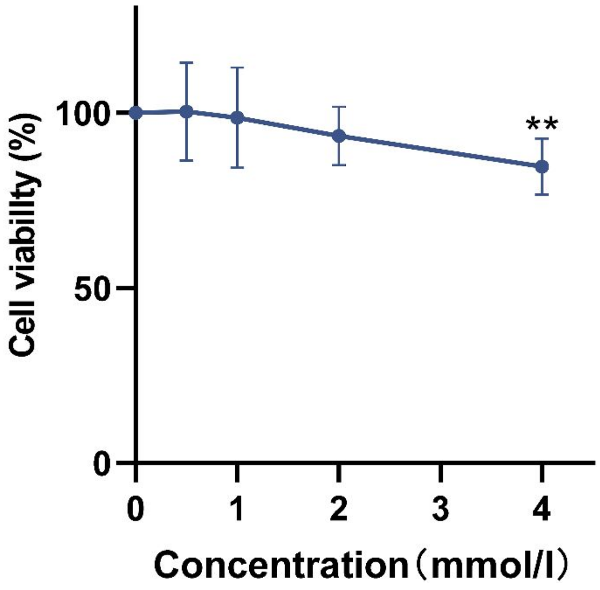

Figure S7. Relative viabilities of mouse VSMCs after incubated with different concentrations of STS (STS concentration: 0-4 mM) (n=5). Data were presented as means ± SD. $^{**}P < 0.01$.

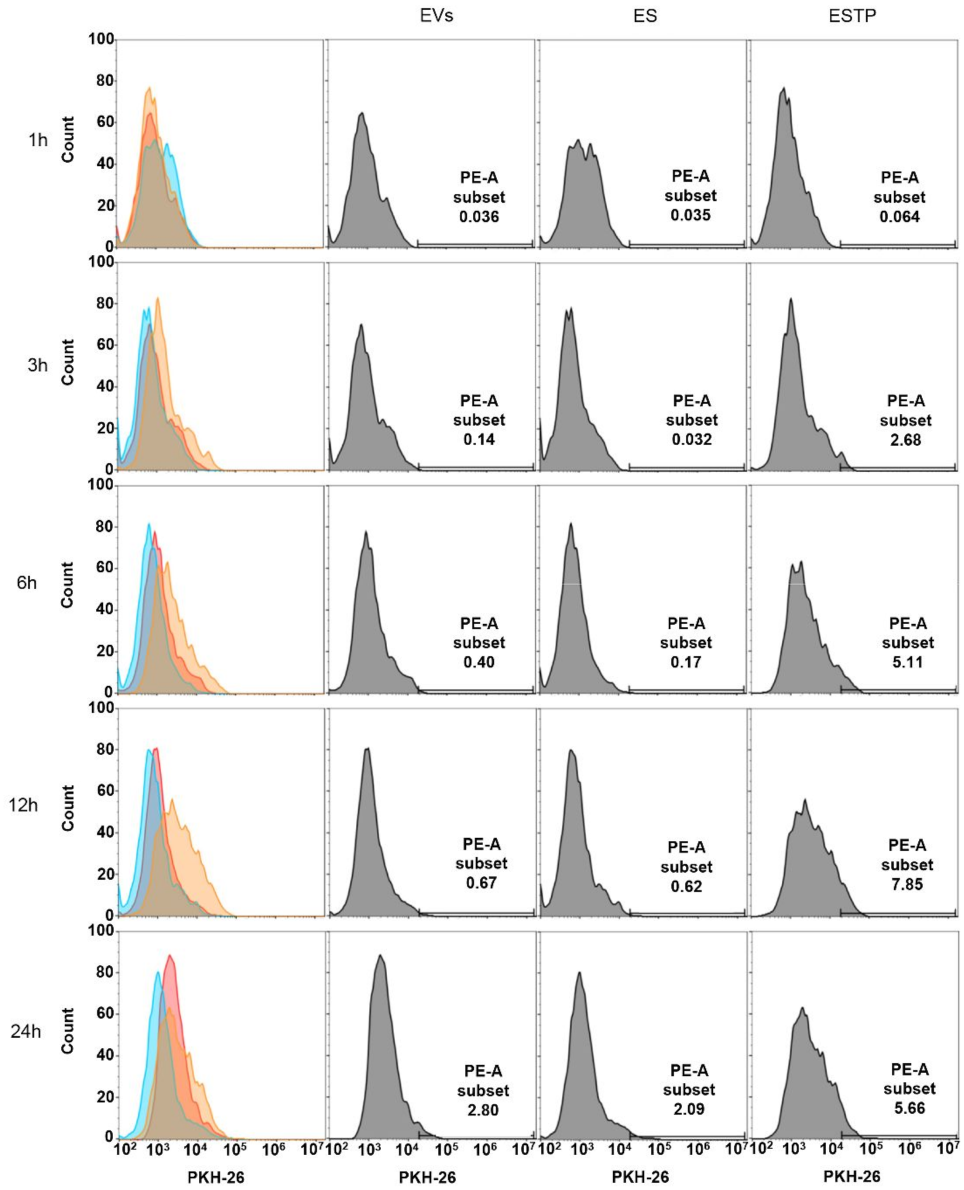

Figure S8. The cellular uptake of PKH26-labeled EVs, ES and ESTP (10 mg L^{-1} EVs) in calcified mouse VSMCs at 1,3,6,12 and 24 hours, as detected by flow cytometry. Results are represented as mean ± SD (n = 3).

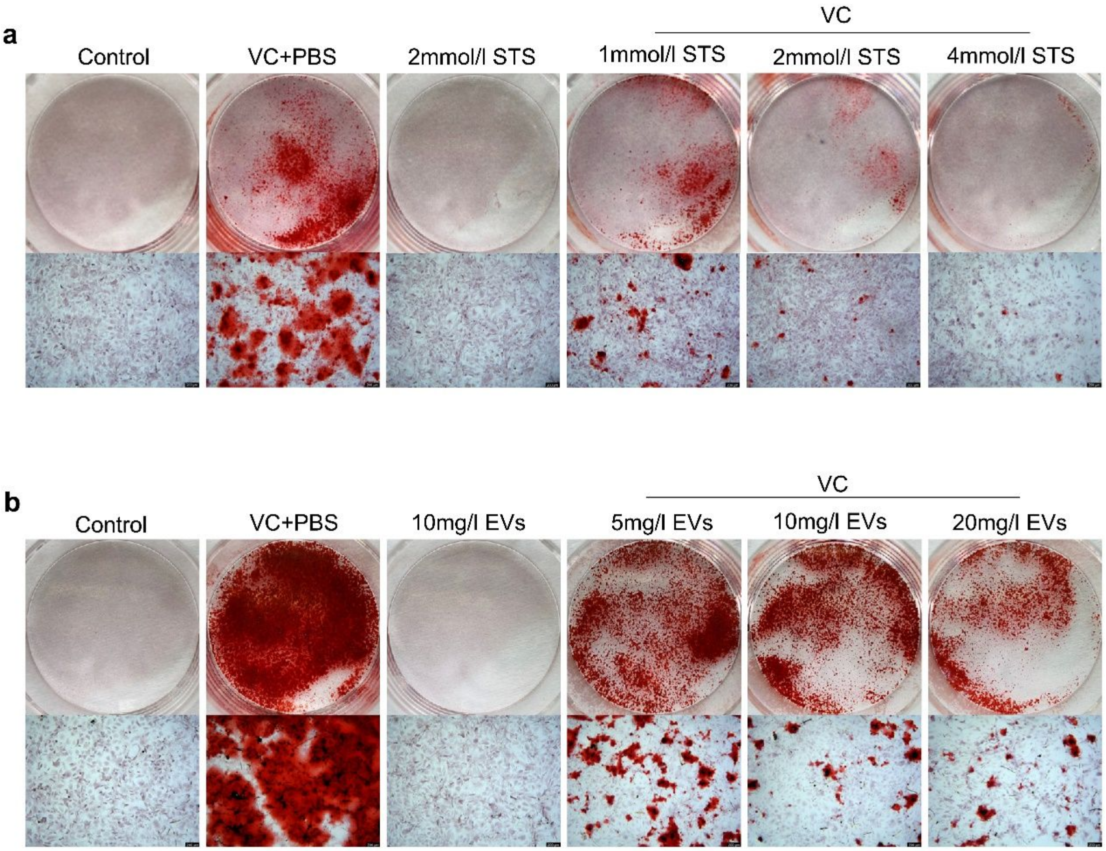

Figure S9. Effects of STS and EVs on calcification of mouse VSMCs. a) Mouse VSMCs were incubated with STS (1, 2 and 4 mM) in the general medium or calcifying medium for 7 days. b) Mouse VSMCs were incubated with EVs (5, 10 and 20 mg·L^{-1}) in the general medium or calcifying medium for 7 days. Mineral deposition in mouse VSMCs was detected by alizarin red staining (n=5). Scale bar: 200μm.

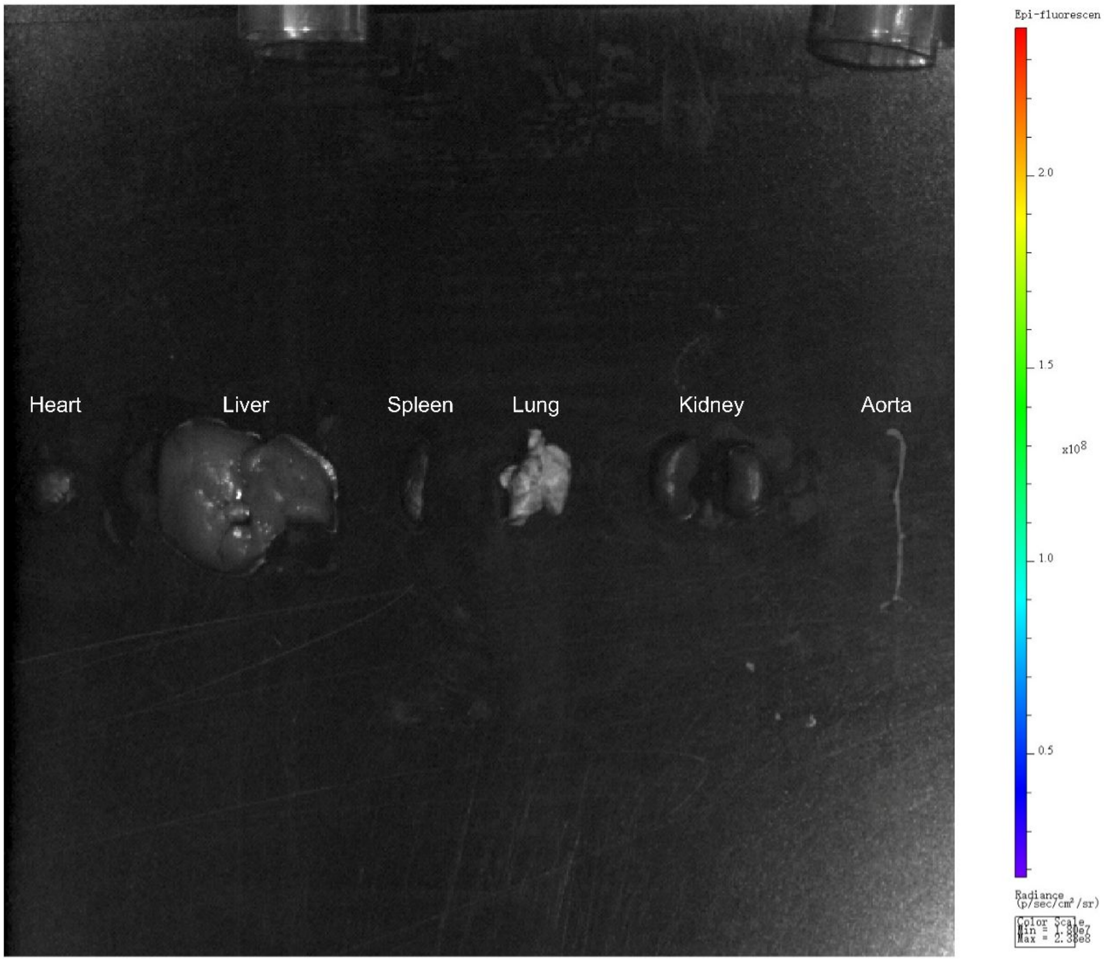

Figure S10. Fluorescence bio-imaging of Cy7 fluorescent signal in aorta tissues and major organs (heart, liver, spleen, lung and kidney) after mice were injected intraperitoneally with PBS.

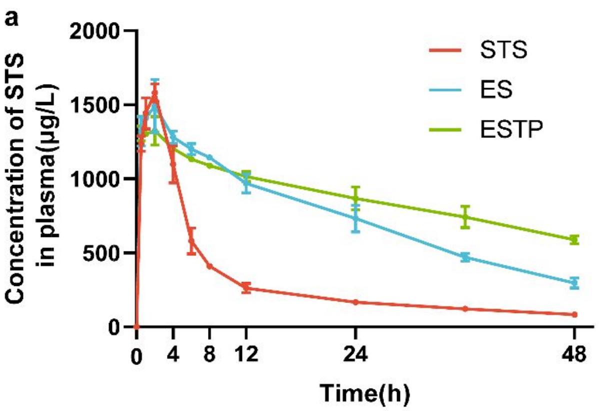

b

	Cmax [ug/L]	CL [L/h/kg]	AUC 0-∞ [ug/L*h]	MRT 0-∞ [h]	t1/2[h]	
					t1/2α [h]	*t1/2β* [h]
**STS**	1645.196	23.538	14862.504	18.320	1.139	20.209
**ES**	1696.794	9.357	42750.952	24.752	1.477	22.590
**ESTP**	1434.773	5.364	74568.550	58.909	6.152	61.748

Figure S11. Pharmacokinetic analysis. a) Plasma concentration-time profiles of STS in VC mice model after intraperitoneal injection of STS, ES and ESTP (50mg kg^{-1} STS). b) The data of pharmacokinetic analysis obtained by DAS 2.0. C_{max}: Maximum concentration; CL: Clearance; AUC: Area under the curve; MRT: Mean residence time; t1/2α and t1/2β: Half-lives in the α and β phases. Data were presented as means ± SD (n=3).

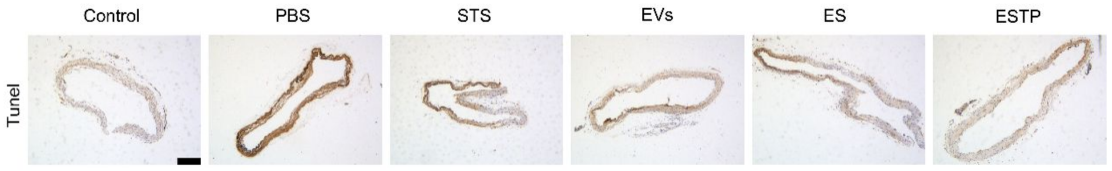

Figure S12. Representative images of terminal deoxynucleotidyl transferase–mediated deoxyuridine triphosphate nick end labeling (TUNEL) staining of aortic arch sections (n = 3). Scale bar: 200μm.

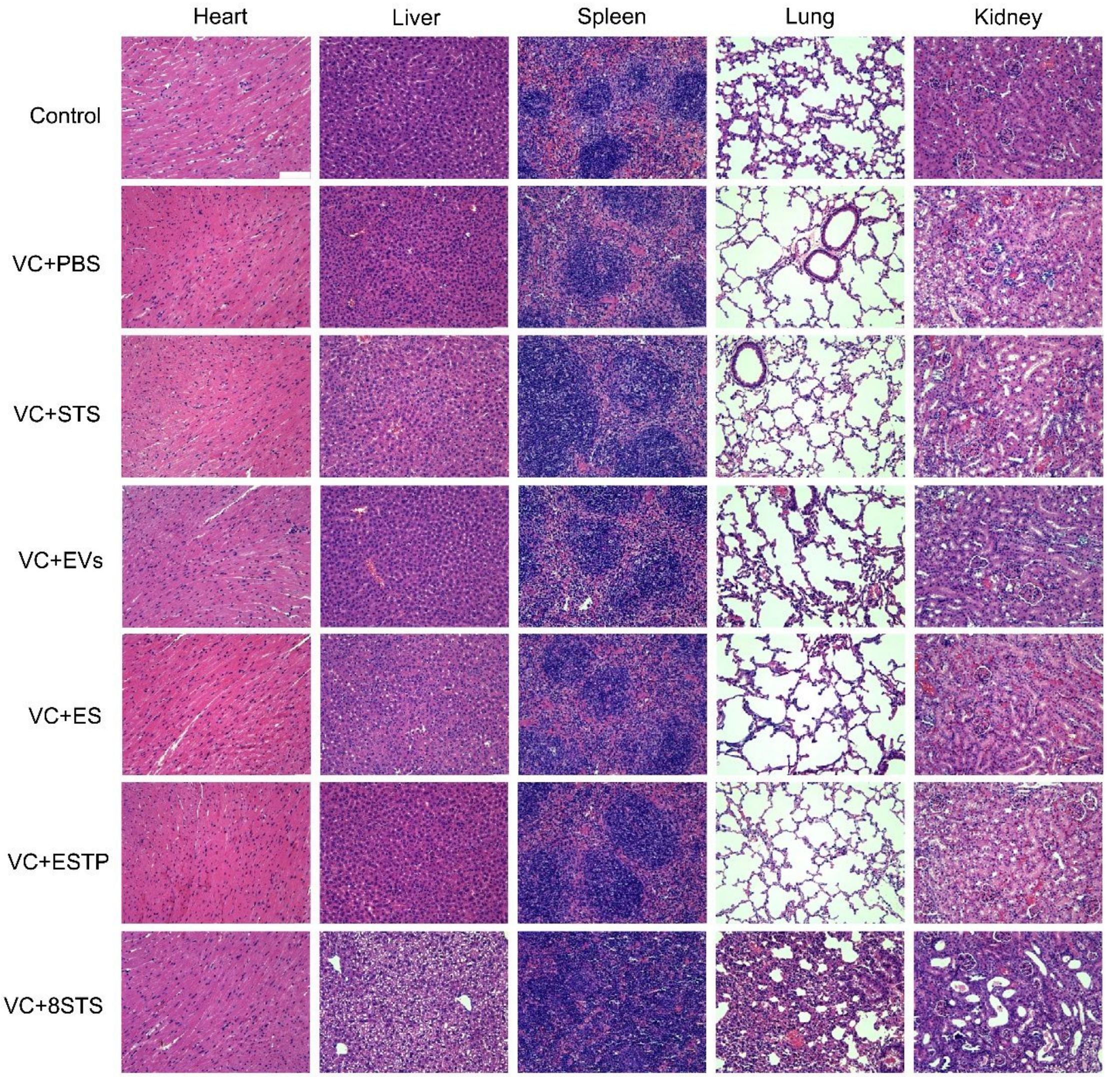

Figure S13. H&E staining of main organs including heart, liver, spleen, lung and kidney collected from mice after different treatments (n=6). Scale bar: 100μm.

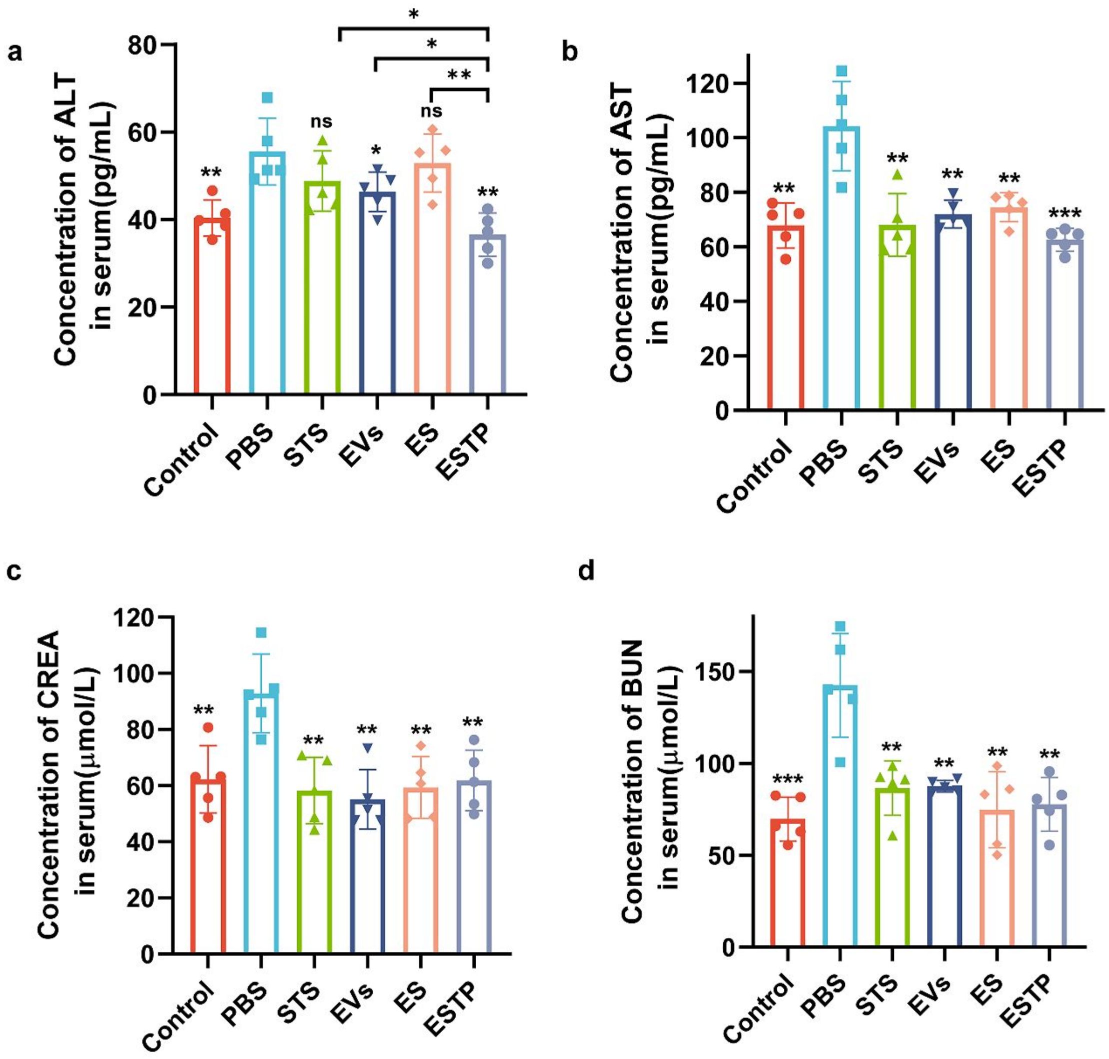

Figure S14. Serum biochemical indicators. Serum levels of a) ALT, b) AST, c) CREA and d) BUN in mice after administration of PBS, STS, ES, ESTP (50 mg kg^{-1} STS) and EVs (equivalently to ESTP) via intraperitoneal injection. ALT, alanine aminotransferase; AST, aspartate aminotransferase; BUN, blood urea nitrogen; Cre, creatinine. Results are represented as mean ± SD (n = 5). $^{*}P < 0.05$, $^{**}P < 0.01$, and $^{***}P < 0.001$.

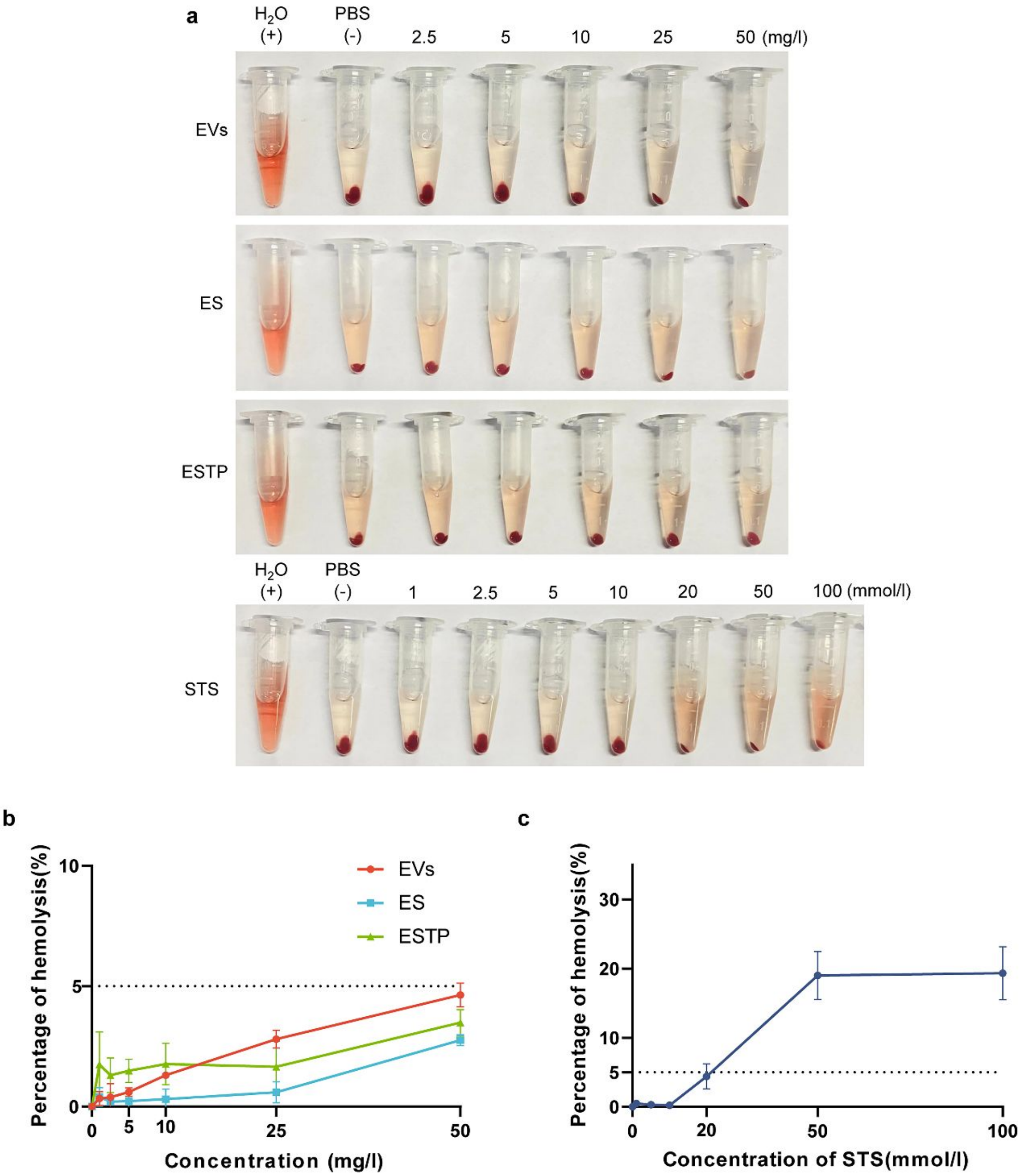

Figure S15. Hemolysis assay. a) The images of red blood cells incubated with EVs, ES, ESTP and STS. b-c) The corresponding OD values at 576 nm of the supernatants were quantified to determinate the magnitude of red blood cell hemolysis. PBS and ultrapure water were set as a negative and positive control, respectively. Results are represented as mean ± SD (n = 5). $^{*}P < 0.05$, $^{**}P < 0.01$, and $^{***}P < 0.001$.

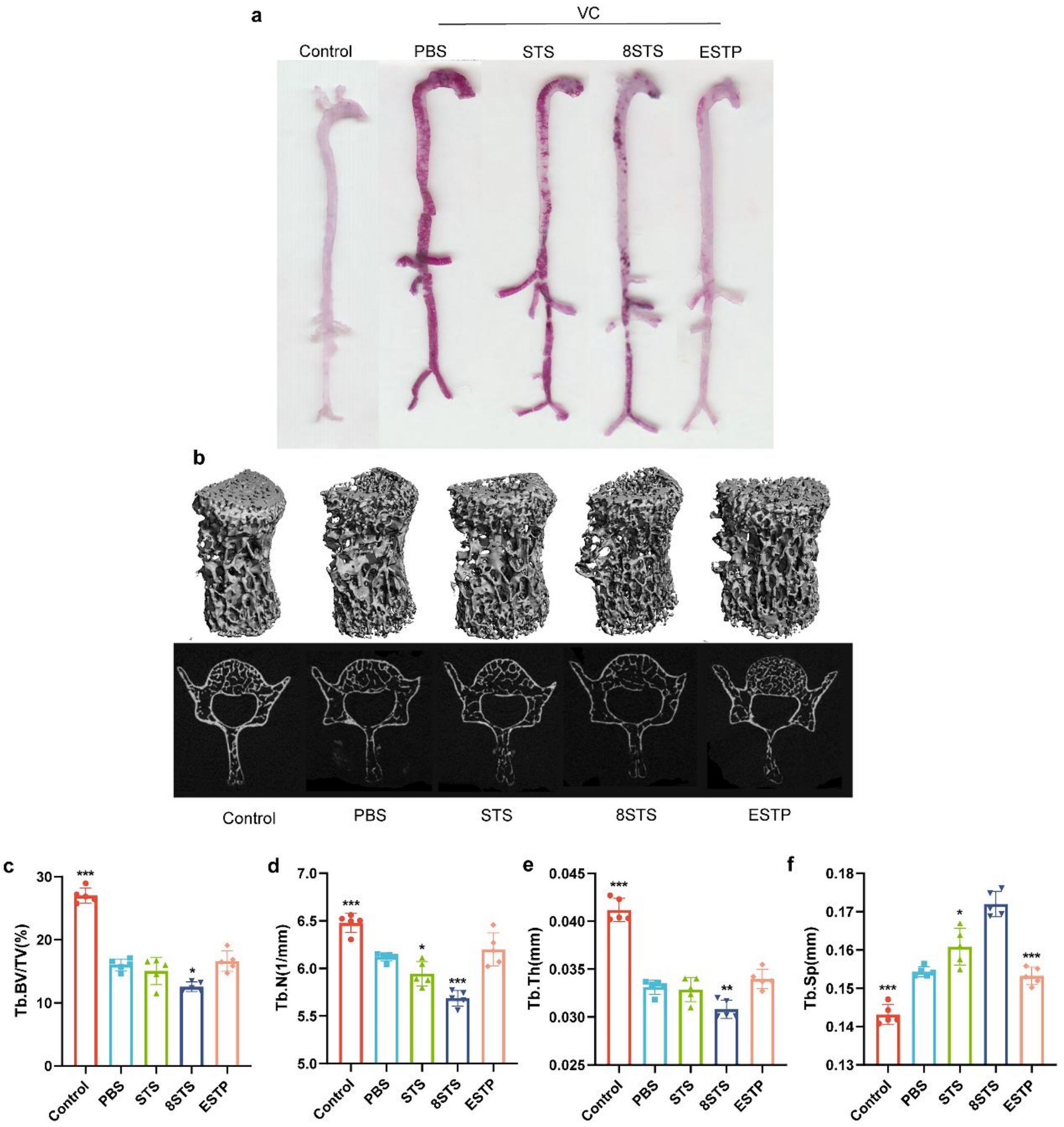

Figure S16. Evaluation of a) anti-VC effect and b-f) bone quality of STS, ESTP (50 mg kg^{-1} STS, an eighth of therapeutic dose for VC as previous studies) and 8STS (400 mg kg^{-1} STS, therapeutic dose for VC as previous studies) in mice. Calcium deposition in aortic arteries was detected by alizarin red staining and bone quality was measured by micro-computed tomography. (n=6). *P* value style: $^{*}P < 0.05$; $^{**}P < 0.01$; $^{***}P < 0.001$.

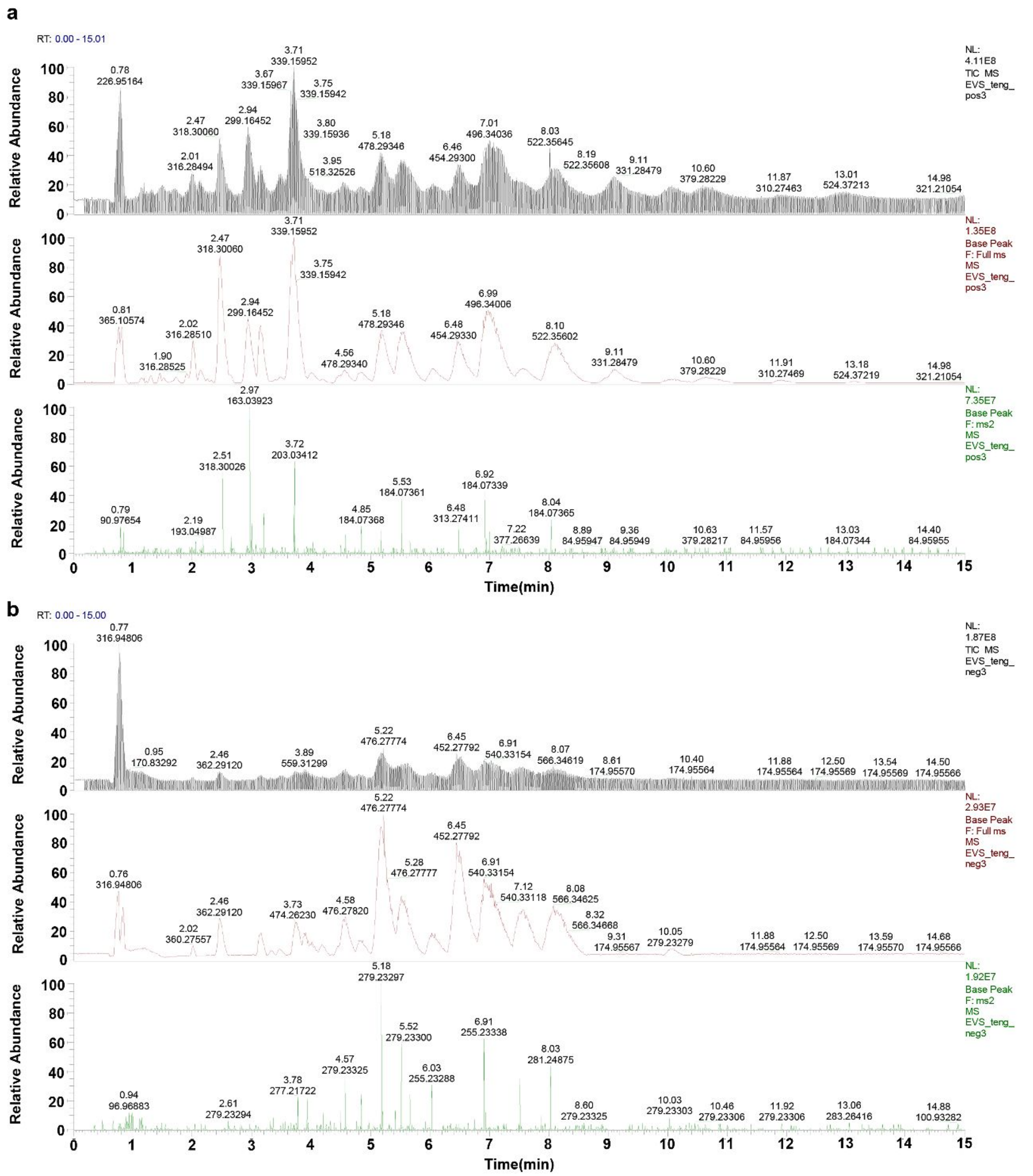

Figure S17. HPLC-MS analysis of the compounds from EVs. a) HPLC-positive and b) HPLC-negative ion ESI-MS total ion current (TIC) profile.

Table S1. A total of 2398 chemical components of grapefruit-derived EVs were identified by HPLC-MS.

Table S2. A total of 27 bioactive components from grapefruit-derived EVs were screened out according to content, bioavailability and drug-likeness

Table S3. Targets and target symbols corresponding to bioactive compounds

Table S4. Interactions of common predicted targets of grapefruit-derived EVs against VC

Table S5. Main biological processes of grapefruit-derived EVs against VC

Table S6. Major regulatory pathways of grapefruit-derived EVs against VC
